# Complete genome sequencing and comparison of two nitrogen-metabolizing bacteria isolated from Antarctic deep-sea sediment

**DOI:** 10.1186/s12864-022-08942-6

**Published:** 2022-10-19

**Authors:** Wenqi Liu, Bailin Cong, Jing Lin, Linlin Zhao, Shenghao Liu

**Affiliations:** 1 School of Advanced Manufacturing, Fuzhou University, Fuzhou, 350108 China; 2 First Institute of Oceanography, Ministry of Natural Resources, Qingdao, 266061 China

**Keywords:** Cobetia amphilecti, Halomonas profundus, Whole genome, Nitrification and denitrification, Inorganic carbon fixation

## Abstract

**Background:**

Bacteria are an essential component of the earth`s biota and affect circulation of matters through their metabolic activity. They also play an important role in the carbon and nitrogen cycle in the deep-sea environment. In this paper, two strains from deep-sea sediments were investigated in order to understand nitrogen cycling involved in the deep-sea environment.

**Results:**

In this paper, the basic genomic information of two strains was obtained by whole genome sequencing. The *Cobetia amphilecti* N-80 and *Halomonas profundus* 13 genome sizes are 4,160,095 bp with a GC content of 62.5% and 5,251,450 bp with a GC content of 54.84%. Through a comparison of functional analyses, we predicted the possible C and N metabolic pathways of the two strains and determined that *Halomonas profundus* 13 could use more carbon sources than *Cobetia amphilecti* N-80. The main genes associated with N metabolism in *Halomonas profundus* 13 are *nar*G, *nar*Y, *nar*I, *nir*S, *nor*B, *nor*C, *nos*Z, and *nir*D. On the contrast, *nir*D, using NH_4_^+^ for energy, plays a main role in *Cobetia amphilecti* N-80. Both of them have the same genes for fixing inorganic carbon: *icd*, *ppc*, *fdh*A, *acc*C, *acc*B, *acc*D, and *acc*A.

**Conclusion:**

In this study, the whole genomes of two strains were sequenced to clarify the basic characteristics of their genomes, laying the foundation for further studying nitrogen-metabolizing bacteria. *Halomonas profundus* 13 can utilize more carbon sources than *Cobetia amphilecti* N-80, as indicated by API as well as COG and KEGG prediction results. Finally, through the analysis of the nitrification and denitrification abilities as well as the inorganic carbon fixation ability of the two strains, the related genes were identified, and the possible metabolic pathways were predicted. Together, these results provide molecular markers and theoretical support for the mechanisms of inorganic carbon fixation by deep-sea microorganisms.

**Supplementary Information:**

The online version contains supplementary material available at 10.1186/s12864-022-08942-6.

## Background

The genus *Cobetia* is classified within the family *Halomonadaceae*, order *Oceanospirillales*, and class *Gammaproteobacteria* within the phylum *Proteobacteria*. The genus is characterized by GRAM-negative, straight, rod-shaped cells of 1.6–4.0 by 0.8–1.2 µm that occur singly and in pairs [1]. Some reports have also revealed that members of the genus *Cobetia* are promising sources of unique enzymes and secondary metabolites, can produce an alkaline phosphatase with unusually high specific activity [2, 3], can synthesize hydroxyectoine under NaCl induction and are tolerant to osmotic stress [4], and have antibiofilm activity [5]. Due to their 16S rRNA sequences with high homology, Noskova suggested that gene-specific oligonucleotides corresponding to the coding sequences for factors with vital bacterial cell functions, such as the alkaline phosphatases PhoA and PhoD may be used for the rapid molecular differentiation of closely related species of the marine bacterial genus *Cobetia* [6]. In addition, cloning and expression have been performed for several of its functional genes, such as *ect*ABC and its promotor sequence in *Cobetia marina* CICC10367 [7], the L-asparaginase gene (CobAsnase) in *Cobetia amphilecti* AMI6 [8], and alkaline phosphatase in *Cobetia marina* [9]. *Cobetia amphilecti* has the ability to remove excess ammonia-N in seawater ponds, removing 61.7% of the total ammonia-N (50 mg/L) in 8 h [10]. However, no analysis was conducted at the genetic level.

The Halomonas was described as a new genus by Vreeland in 1980. In 1996, Dobson & Franzmann proposed that members of the genera *Deleya*, *Halomonas*, and *Halovibrio* should be placed in the genus *Halomonas* within the phylum *Proteobacteria*. The genus *Halomonas* was described as a facultative anaerobe with gram-negative, straight or curved rodlike cells [11, 12]. *Halomonas* is distributed in Lake Pengyanco on the Tibetan Plateau [13], the Bohai Gulf of the Yellow Sea in China [14], the Pentha beach of Odisha in India [15], Urmia Lake in Iran and other places [16]; it grows in Gobi soil [17], salt lake sediment [18], the liquid in the stems of *Populus euphratica* [19], the rhizosphere sand of a coastal sand dune plant [20] and other environments, with slight or moderate halophily. Among *Halomonas* sp., *Halomonas bluephagenesis* is a relatively comprehensively studied species with engineering tools and methods for genetic modification available. Due to its potential for use in contamination treatment, it can be grown under open and continuous processes not only in the lab but also at an at least 1000 L bioreactor scale [21]. To date, many studies have explored its potential for the production of L-threonine [22], starch [23], 3-hydroxypropionate [24], functional polyhydroxyalkanoates [25], bioplastic PHB and ectoine [26]. *Halomonas* can grow under high salt concentrations at alkaline pH and can resist contamination by other microbes, so it has good prospects for various applications.

*Cobetia* and *Halomonas* belong to the *Halomonadaceae* family. According to the genome information uploaded in NCBI, there are currently 16 genera of *Halomonadaceae* family, among which *Cobetia* and *Halomonas* contain more species. Most of *Halomonadaceae* are moderately halophilic bacteria, and they often have high abundance in some places with high salt content such as surface seawater [27], saline agricultural soil [28] and surface sediments [29]. Prokaryotes populate every habitable environment on Earth and affect the chemistry and physical properties of their surroundings through their metabolic activity [30]. Thus, microbes are dominant drivers of biogeochemical processes [31] and have probably even determined the basic composition of Earth’s atmosphere since the origin of life [32]. The ocean microbiome is a highly dilute microbial system that covers the majority of the Earth’s surface and extends an average of 3600 m down to the seafloor [33]. These ocean microbes are responsible for half of all primary production occurring on Earth [34] and play key roles in ocean carbon and nutrient cycling [31, 35]. Swan proposed that unidentified prokaryotes fix inorganic carbon at globally significant rates in the immense dark ocean [36]. The pelagic realm of the dark ocean was reported to represent a key site for the remineralization of organic matter and for long-term carbon storage and burial in the biosphere [37]. The dark ocean below 200 m comprises approximately 75% of global oceanic volume and contains more than 98% of the global dissolved inorganic carbon pool [38].

At present, the known marine microorganisms that play an important role in fixing inorganic carbon are Thaumarchaeota [39], *Nitrosopumilus maritimus* [40], *Nitrospira-*like bacteria [41], and *Nitrospira marina* [42]. The fixation of inorganic carbon by these microorganisms is mostly coupled with ammonia oxidation, nitrification and other reactions. According to the plankton average C/N/P ratio (106:16:1) [43], the nitrogen flux released by downwardly deposited particulate organic matter should be very large [44]. Nitrogen can be ammoniated by microorganisms to produce ammonium. Nonetheless, once ammonium is formed, in the presence of molecular oxygen, it is oxidized by nitrifying bacteria to form nitrite and nitrate [45]. Nitrifying bacteria harvest the chemical energy stored in NH_4_^+^ and fix CO_2_ to synthesize the organics they need. In the absence of oxygen, NO_3_^−^ can be used by many microbes as a respiratory electron acceptor, and at the same time, nitrate reduction is coupled to the anaerobic oxidation of organic carbon [46]. An article reported that ammonia oxidation to nitrite and its subsequent oxidation provided energy to the two populations of nitrifying chemoautotrophs in the energy-starved dark ocean, driving a coupling of reduced inorganic nitrogen pools and the production of new organic carbon in the dark ocean [47]. Current research shows that deep-sea microbial nitrification serves as an important energy source in deep-sea ecosystems by fixing inorganic carbon through chemical energy autotrophy, which even directly affects the food network structure of the deep-sea ecosystem and carbon storage [44, 48]. Therefore, microorganisms can utilize ammonium nitrogen and nitrite oxidation to provide electrons and energy for nitrifying bacteria to fix inorganic carbon, which provides theoretical support for understanding marine carbon storage and enriches the theoretical basis of the nutritional structure in deep-sea ecosystems.

Dissolved organic carbon produced by microorganisms through carbon sequestration can also be further converted into inert dissolved organic carbon by microbial carbon pumps and stored in the deep sea, which can realize the long-term storage of carbon dioxide and influence the carbon flux in the deep sea. As described previously, Carbon metabolism is highly coupling with nitrogen which few studies have been paid. After separation and purification of marine sediment samples, two strains were obtained by functional screening, which could grow on nitrification and denitrification medium. Through the preliminary query of strains information, we found that two kinds of strains has been not studies regarding nitrogen metabolism, which could be valuable for deeply understanding the regulation of nitrogen cycle in deep sea. We sequenced their whole genomes separately to obtain their basic genomic information for subsequent analysis and to provide a molecular basis for future studies of the two strains. Then, we predicted the relevant metabolic pathways to provide molecular markers and theoretical support for studying biological nitrogen metabolism in ecosystems.

## Results

### Strain properties and phylogeny

**Fig. 1:**
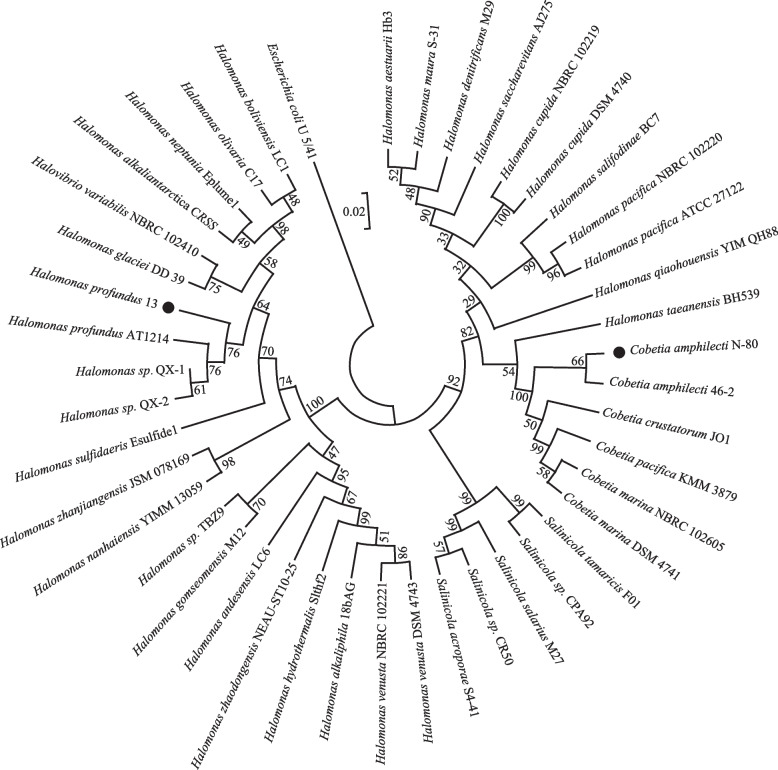
Phylogenetic tree produced by the comparison of the 16S rRNA sequences. The *Cobetia amphilecti* N-80 and the *Halomonas profundus* 13 are represented with black dots. Numbers on the nodes are bootstrap values in percentage (1000 replicates)

The two strains that were studied in this paper were isolated from deep sea marine sediments collected during the 34th Chinese National Antarctica Expedition. The sampling sites were all in the waters off the Antarctic and the southern end of the Atlantic Ocean in the Scotia Sea. We screened two strains that could grow on both nitrifying and denitrifying selective medium, namely, N-80 and 13. N-80 exhibited optimum growth at 15 °C (range, 15–37 °C) and 2–12% NaCl (w/v; optimum 4% NaCl), and 13 exhibited optimum growth at 37 °C (range, 15–37 °C) and 2–18% NaCl (w/v; optimum 8% NaCl). The 16S rRNA sequencing results yielded effective fragment lengths of 1300–1400 bp for both strains, which were identified as *Cobetia amphilecti* and *Halomonas profundus* according to the similarity via BLAST on NCBI. A phylogenetic tree was constructed by neighbor-joining (NJ) method based on the 16S rRNA sequences using *E. coli* as an outgroup genus and with a bootstrap value of 1000 (Fig. 1). And it confirmed by Maximum Likelihood (ML) method (Fig. S1).

The 16S rRNA of N-80 is highly similar to that of *Cobetia amphilecti*. Whole-genome sequences of two strains of *Cobetia amphilecti* have been downloaded on the NCBI, *Cobetia amphilecti* B2M13 (NZ_JAHKQM000000000) and *Cobetia amphilecti* KMM296 (NZ_JQJA00000000). To understand the physiological and biochemical patterns among the strains, API 20NE reagent strips were used to analyze the two strains. The results showed that *Cobetia amphilecti* N-80 had urea, β-glucosidase, and β-galactosidase. *Halomonas profundus* 13 tested positively on reduction capacities for nitrate to nitrite or nitrogen and was able to acidify glucose; this strain also contained arginine dihydrolase, urea, β-glucosidase, and β-galactosidase. The carbon sources that could be used were glucose, arabinose, mannitol, maltose, gluconate, capric acid, adipic acid, malic acid, citric acid, and phenylacetic acid.

### General features of the genome

The complete genomes of the two strains were sequenced by using Illumina and Nanopore sequencing technology to generate single contigs for each strain. The genomic traits of the two strains can be found in Table 1. The average sequencing depth of *Cobetia amphilecti* N-80 was 434.58 × and 237.11 × for the second-generation and third-generation-sequencing, respectively. The average sequencing depth of *Halomonas profundus* 13 was 308.81 × and 188.36 × for the second-generation and third-generation sequencing, respectively. The *Cobetia amphilecti* N-80 genome size is 4,160,095 bp with 62.5% GC content. The *Halomonas profundus* 13 genome size is 5,251,450 bp with 54.84% GC content. The complete genome sequences of *Cobetia amphilecti* N-80 and *Halomonas profundus* 13 were deposited in the NCBI database under accession numbers NC_CP084115 and CP086344. The predicted genome information, such as genome sequencing depth, GC distribution and genome structure annotation, was integrated to draw circular genome maps as shown in Figure S2 (a) and Figure S2 (b) for *Cobetia amphilecti* N-80 and *Halomonas profundus* 13, respectively.

**Table 1:** The result of average nucleotide identity (ANI) analysis

	Halomonas profundus 13	Halomonas olivaria TYRC17	Halomonas sulfidaeris ATCC BAA-803	Halomonas axialensis Althf1	Halomonas hydrothermalis Slthf2	Halomonas piezotolerans NBT06E8	Halomonas subglaciescola ACAM 12	Halomonas huangheensis BJGMM-B45
Halomonas profundus 13	*	87.52 [72.27]	82.73 [58.15]	77.20 [47.31]	76.69 [51.47]	76.08 [52.04]	73.36 [32.08]	70.20 [33.86]
Halomonas olivaria TYRC17	87.59 [75.50]	*	82.20 [59.66]	77.25 [49.01]	76.28 [51.55]	76.26 [53.78]	73.63 [33.02]	70.37 [34.69]
Halomonas sulfidaeris ATCC BAA-803	83.02 [71.30]	82.28 [70.17]	*	76.15 [53.53]	75.52 [55.10]	75.34 [59.64]	72.78 [38.07]	70.19 [36.67]
Halomonas axialensis Althf1	77.42 [67.78]	77.14 [67.19]	76.13 [62.60]	*	78.32 [68.11]	86.01 [76.75]	73.90 [45.34]	70.93 [39.57]
Halomonas hydrothermalis Slthf2	76.94 [65.16]	76.46 [61.83]	75.53 [56.90]	78.48 [60.20]	*	77.21 [62.65]	72.17 [40.69]	69.67 [37.66]
Halomonas piezotolerans NBT06E8	76.20 [68.89]	76.15 [67.68]	75.29 [63.80]	85.84 [70.26]	77.17 [65.05]	*	73.66 [41.59]	70.84 [39.68]
Halomonas subglaciescola ACAM 12	73.76 [53.10]	73.86 [51.86]	73.09 [50.59]	74.38 [53.81]	72.77 [53.52]	74.15 [52.72]	*	71.69 [43.66]
Halomonas huangheensis BJGMM-B45	70.16 [37.60]	70.36 [35.97]	69.95 [32.62]	70.75 [29.97]	69.65 [32.46]	70.71 [33.06]	71.31 [29.21]	*
	Cobetia amphilecti N-80	Cobetia amphilecti KMM296	Cobetia amphilecti B2M13	Cobetia marina JCM 21,022	Cobetia crustatorum SM1923	Cobetia pacifica GPM2	Cobetia sp. L2A1	Cobetia sp. MB87
Cobetia amphilecti N-80	*	96.78 [88.29]	96.50 [90.02]	86.54 [74.45]	81.42 [67.06]	86.92 [75.48]	81.23 [67.17]	93.83 [68.45]
Cobetia amphilecti KMM296	96.73 [92.61]	*	96.26 [91.96]	86.23 [75.71]	81.36 [69.51]	86.74 [77.84]	80.92 [68.68]	80.92 [68.68]
Cobetia amphilecti B2M13	96.31 [87.06]	96.03 [84.98]	*	86.60 [75.58]	81.24 [66.01]	86.58 [74.82]	81.21 [66.81]	93.66 [67.43]
Cobetia marina JCM 21,022	86.56 [74.36]	86.34 [71.89]	86.84 [77.63]	*	81.38 [70.73]	97.97 [92.39]	80.84 [69.76]	86.36 [60.71]
Cobetia crustatorum SM1923	81.16 [66.36]	81.22 [65.51]	81.17 [67.44]	81.03 [70.11]	*	80.91 [69.99]	85.28 [78.66]	80.89 [54.31]
Cobetia pacifica GPM2	86.83 [75.45]	86.74 [74.05]	86.72 [76.77]	97.80 [92.34]	81.11 [70.43]	*	80.50 [68.46]	86.31 [60.88]
Cobetia sp. L2A1	81.12 [67.70]	80.98 [65.39]	81.25 [69.31]	80.77 [69.78]	85.53 [80.58]	80.51 [68.84]	*	80.84 [53.41]
Cobetia sp. MB87	94.66 [90.40]	94.38 [87.59]	94.64 [92.39]	86.72 [79.34]	81.31 [71.67]	86.76 [79.78]	81.03 [70.25]	*

^a^Halomonas profundus 13

^b^Cobetia amphilecti N-80

We compared the whole-genome sequences of these strains with those of *Cobetia amphilecti* N-80 and *Halomonas profundus* 13. ANI analysis showed similarity values of 96.50% (*Cobetia amphilecti* N-80 and *Cobetia amphilecti* B2M13) and 96.78% (*Cobetia amphilecti* N-80 and *Cobetia amphilecti* KMM296) (Table 2). The results revealed that *Cobetia amphilecti* N-80 should be considered the same species as *Cobetia amphilecti* B2M13 and KMM296, as the ANI similarity was greater than 95%. The highest similarity to 13 was found for *Halomonas olivaria* TYRC17, with a similarity of 87.52%. *Cobetia amphilecti* N-80 performed AAI and DDH analysis with *Cobetia amphilecti* KMM296 and *Cobetia amphilecti* B2M13 respectively. The results showed that the AAI of *Cobetia amphilecti* N-80, *Cobetia amphilecti* KMM296 and *Cobetia amphilecti* B2M13 were 98.06% and 97.95%, respectively, which were all greater than 95%. The DDH of *Cobetia amphilecti* N-80, *Cobetia amphilecti* KMM296 and *Cobetia amphilecti* B2M13 was 74.2% and 71.8%, respectively, which were all greater than 70%. From the above data, *Cobetia amphilecti* N-80, *Cobetia amphilecti* KMM296 and *Cobetia amphilecti* B2M13 are the same species. But the downloaded genome information is incomplete.

**Table 2:** The obtained genomic information of *Cobetia amphilecti* KMM296 and *Cobetia amphilect*i B2M13 was compared with that of *Cobetia amphilecti* N-80

	Cobetia amphilecti N-80	Cobetia amphilecti KMM296	Cobetia amphilecti B2M13
Assembly Level	Complete	Contig	scaffold
Length of genome assembly(Mb)	4.16	3.97	4.29
GC%	62.5	62.5	62.5
Gene	3,481	3.401	3,573
CDS	3,324	3,271	3,485
Number of scaffolds	1	97	27
contigs	1	97	58
N50	4,160,095	89,168	123,498
L50	1	14	10

**Fig. 2:**
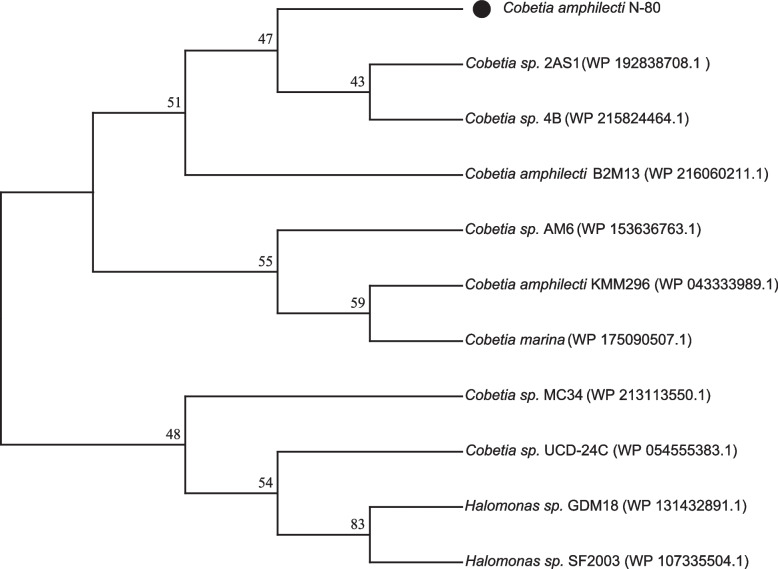
Phylogenetic neighbor-joining tree of PhoD proteins. The *Cobetia amphilecti* N-80 is represented with black dots. Numbers on the nodes are bootstrap values in percentage (1000 replicates)

When we reviewed the uploaded genomic information of *Cobetia amphilecti* on NCBI, we found that *Cobetia amphilecti* KMM296 had 97 scaffolds and *Cobetia amphilecti* B2M13 had 27 scaffolds. Subsequently, the obtained genomic information of *Cobetia amphilecti* KMM296 and *Cobetia amphilecti* B2M13 was compared with that of *Cobetia amphilecti* N-80, and the results are shown in Table 3. Next, we constructed a neighbor-joining tree using the gene-specific oligonucleotide *pho*D, corresponding to the DNA sequence that encodes an enzyme responsible for important functions of bacterial cells in the genus *Cobetia* (Fig. 2). Meanwhile the Maximum likelihood tree of PhoD was constructed (Fig. S3). They could be concluded that *Cobetia amphilecti* N-80, *Cobetia amphilecti* B2M13 and *Cobetia amphilecti* KMM296 are different strains of the same species.

**Table 3:** Genome properties of the strains sequenced in this study

	Cobetia amphilecti N-80	Halomonas profundus 13	software
Sequence Length	4,160,095	5,251,450	Unicycler(0.4.9)
GC%	62.5%	54.84%	
Genes	3504	4842	Prokka(1.13)
CDS	3386	4727	
5S rRNAs	7	6	RNAmmer(1.2)
16S rRNAs	7	6	
23S rRNAs	7	6	
tRNAs	76	61	Aragorn(v1.2.38)
tmRNA	1	1	
misc RNA	20	35	Infernal(1.1)
Pseudo Genes	213	338	Pseudofinder
NCBI RefSeq	NC_CP084115	CP086344	NCBI

The genomes were annotated through multiple databases. Of all the genes in the *Cobetia amphilecti* N-80 and *Halomonas profundus* 13 genomes, at least 95.89% and 97.11% were annotated. The COG (Cluster of Orthologous Groups of proteins) is a database that classifies possible gene functions. The results of COG annotation for *Cobetia amphilecti* N-80 and *Halomonas profundus* 13 are shown in Figure S4 and showed that C (energy production and conversion), E (amino acid transport and metabolism), and J (translation, ribosomal structure and biogenesis) are the three most abundant gene types in both strains, with more than 200 gene dosages, indicating that these are essential processes closely related to the life activities of these strains. In contrast, there were 68 and 61 more kinds of genes related to G (carbohydrate transport and metabolism) and N (cell motility) in *Halomonas profundus* 13 than in *Cobetia amphilecti* N-80.

Based on the whole-genome information, we used KEGG (Kyoto Encyclopedia of Genes and Genomes) [49] to predict the most important cellular processes, environmental information processing, genetic information processing, metabolism, and organismal systems (Fig. S5). The KEGG annotation results show that a large proportion of the annotated genes belong to the function of metabolism, indicated by the blue blocks. Among them, the most genes were related to carbohydrate metabolism and amino acid metabolism. At the same time, we found that *Halomonas profundus* 13 possessed 109, 94 and 61 more kinds of genes related to carbohydrate metabolism, membrane transport and cellular activity than *Cobetia amphilecti* N-80. This was consistent with the previous API results. *Halomonas profundus* 13 could have a stronger ability to use different carbon sources than *Cobetia amphilecti* N-80.

### Functional profiling

**Fig. 3:**
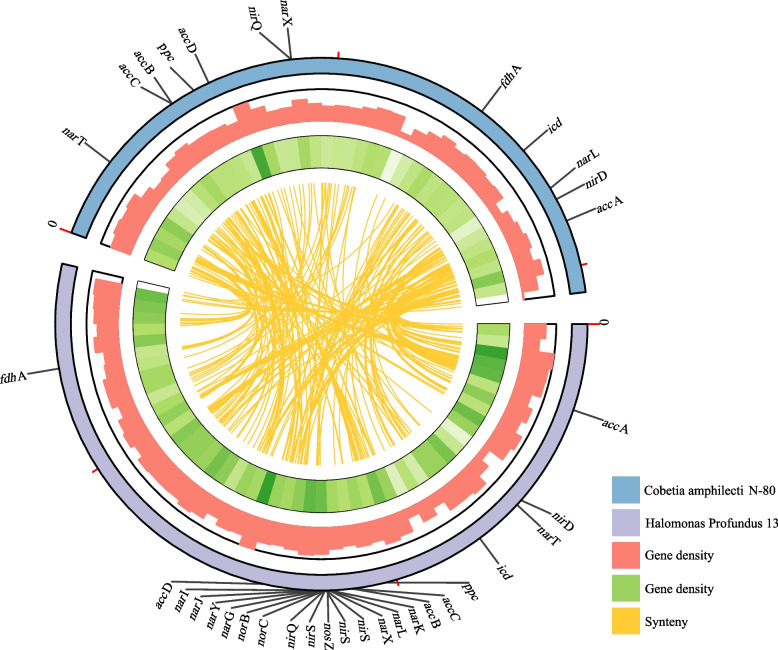
Circos map of the collinearity analysis based on protein-coding genes. The *Cobetia amphilecti* N-80 and the *Halomonas profundus* 13 are shown in blue and purple, respectively. The depth of green and the height of red indicated the gene density, and the yellow was the synteny

**Fig. 4:**
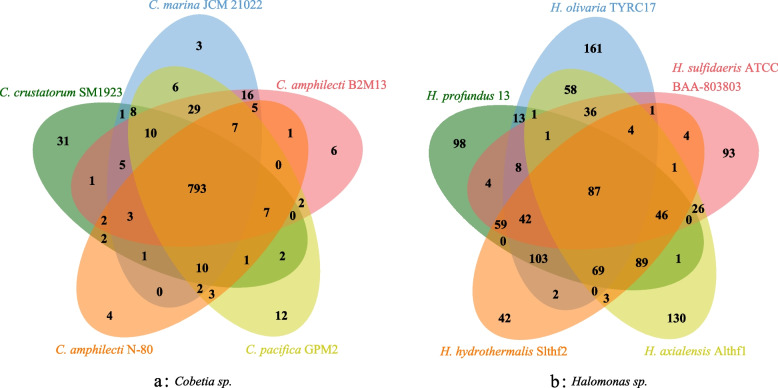
Venn graphs of comparison between the same genera. **a** is the genus of *Cobetia* and **b** is the genus of *Halomonas*. Different colors represent different species

To determine the genomic collinearity between the two strains, we compared their genomes using TBtools to visualize the relationships and the location information for similar segments. Comparative genomic circos map of *Halomonas profundus* 13 and *Cobetia amphilecti* N-80 were drawn and showed that the two genera are closely related (Fig. 3). The two strains had 494 sequences with high homology (E-value less than 10^–5^). The longest such sequence was 12,901 bp, and the shortest one was only 33 bp, but there were relatively few long fragments; only 39.27% of the similar fragments were longer than 1000 bp, and only 3.23% were longer than 3000 bp. The comparison data of the two genomes are shown in Supplementary Table 1. The *Cobetia amphilecti* N-80 and *Halomonas profundus* 13 were compared with other strains of the same genus to further understand the similarities and differences between the same genus. The gene annotation information of the strains was downloaded from NCBI, and the differences between them were shown by Venn diagram. As shown in Fig. 4, a is *Cobetia sp.* and b is *Halomonas sp.*. The *Cobetia sp.* has 793 genes in common; only 87 shared genes in *Halomonas sp.*. *Cobetia amphilecti* N-80 has only four unique genes: lptE, drmD, cadR, sodX; *Halomonas profundus* 13 has more unique function genes.

**Fig. 5:**
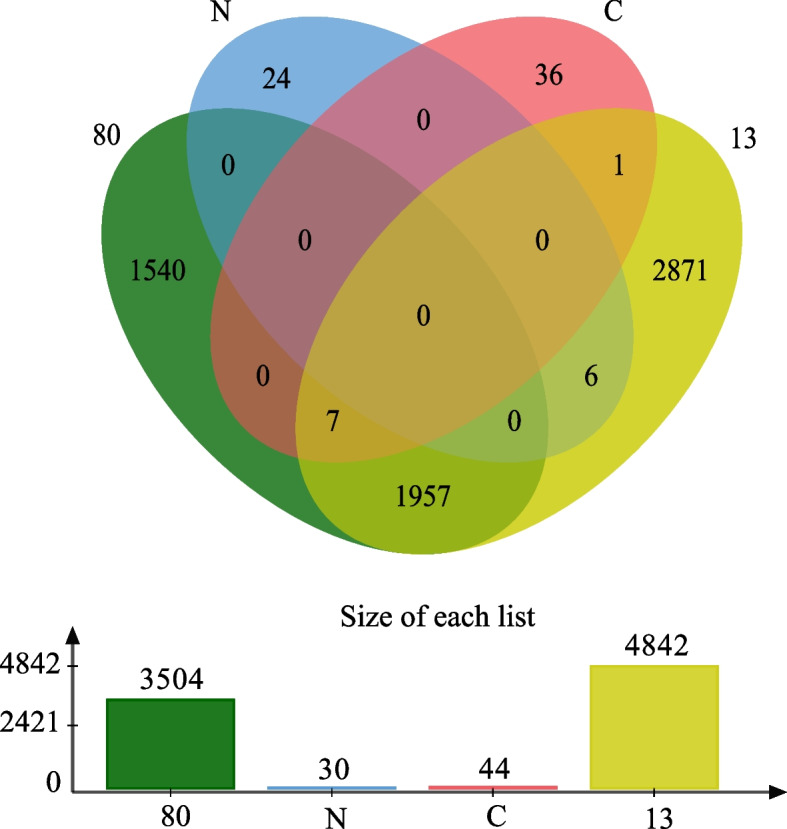
Venn diagram of the number of shared and unique proteins. The genes of the *Cobetia amphilecti* N-80 and the *Halomonas profundus* 13 are shown in green and yellow, respectively. The gene of nitrification and denitrification is shown in blue, and the genes related to fixing inorganic carbon in prokaryotic carbon fixation is shown in red

**Fig. 6:**
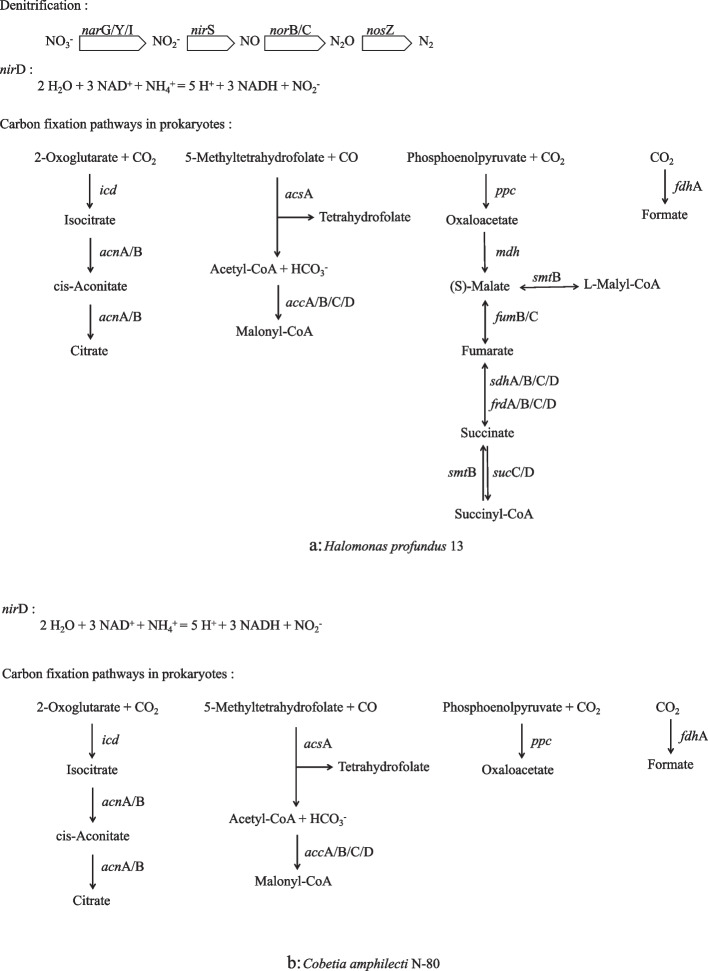
The predicted metabolic pathways. The a is the metabolic pathways of *Halomonas profundus* 13, include denitrification, use NH_4_^+^ and Carbon fixation pathways in prokaryotes; The b is the metabolic pathways of *Cobetia amphilecti* N-80, include use NH_4_^+^ and Carbon fixation pathways in prokaryotes

A total of 30 genes related to nitrification and denitrification (N) and 44 genes related to fixing inorganic carbon in prokaryotic carbon fixation (C) were found in the KEGG and GO databases. The annotation information of the *Halomonas profundus* 13 (4842) and *Cobetia amphilecti* N-80 (3504) genomes were used together with N (30) and C (44) metabolism-related genes to make the Venn diagram (Fig. 5). Figure 5 shows that *Cobetia amphilecti* N-80 and *Halomonas profundus* 13 contain 1964 genes that encode the same proteins, 7 of which are associated with prokaryotic fixation of inorganic carbon, namely, *icd*, *ppc*, *fdh*A, *acc*C, *acc*B, *acc*D, and *acc*A. The *smt*B gene, associated with prokaryotic fixation of inorganic carbon, was not found in *Cobetia amphilecti* N-80 and is present in *Halomonas profundus* 13 alone. No genes associated with nitrification and denitrification were found in *Cobetia amphilecti* N-80 among two databases, and six were found in *Halomonas profundus* 13, specifically, *nar*G, *nar*Y, *nar*I, *nos*Z, *nor*B, and *nor*C. In addition, we found five other genes in both *Halomonas profundus* 13 and *Cobetia amphilecti* N-80 that might be associated with N metabolism, namely, *nar*T (putative nitrate transporter NarT), *nar*X (nitrate/nitrite sensor protein NarX), *nar*L (nitrate/nitrite response regulator protein NarL), *nir*Q (denitrification regulatory protein NirQ), and *nir*D (nitrite reductase (NADH) small subunit). Therefore, in *Cobetia amphilecti* N-80, there are 7 genes related to prokaryotic fixation of inorganic carbon and 5 genes related to nitrification–denitrification; in comparison, *Halomonas profundus* 13 contains 8 genes related to prokaryotic fixation of inorganic carbon and 14 genes related to nitrification–denitrification. The positions of these genes are marked in the circos map (Fig. 3) and the specific information of these genes is in Supplementary Table 2. Some genes encoding regulatory proteins related to nitrogen metabolism that are shared by *Halomonas profundus* 13 and *Cobetia amphilecti* N-80 are shown in Table 4. Based on the proteins, we predicted the possible *Halomonas profundus* 13 (Fig. 6a) and *Cobetia amphilecti* N-80 (Fig. 6b) nitrification, denitrification pathways and pathways associated with the fixation of inorganic carbon. In order to further determine the function of these genes, we compared the protein sequences translated by these genes (Fig. S6), and found that the protein sequences of these enzymes were conserved among different species and genera.

**Table 4:** Shared genes encoding regulatory proteins related to nitrogen metabolism

gene	function
narT	putative nitrate transporter NarT
narX	Nitrate/nitrite sensor protein NarX
narL	Nitrate/nitrite response regulator protein NarL
narK	Nitrate/nitrite transporter NarK
narJ	Nitrate reductase molybdenum cofactor assembly chaperone NarJ
nirQ	Denitrification regulatory protein NirQ

## Discussion

### Comparison between the same genera

We identified strain N-80 as *Cobetia amphilecti* using 16S rRNA sequencing. However, species identification within this clade is complicated due to the high level of identity of their 16S rRNA genes. However, of these, we found only the gene segment 641,535–643,262 encoding alkaline phosphatase D in *Cobetia amphilecti* N-80. Five gene segments encoding alkaline phosphatases were found in *Cobetia amphilecti* B2M13, of which segment 60,707–62,302 encodes an alkaline phosphatase D family protein, one segment encodes an alkaline phosphatase, and three others encode alkaline phosphatase family proteins. Five segments of genes encoding alkaline phosphatase were also identified in *Cobetia amphilecti* KMM296, of which segment 55,751–57,346 encodes an alkaline phosphatase D family protein, one segment encodes an alkaline phosphatase, and three others encode alkaline phosphatase family proteins. We constructed a phylogenetic evolutionary tree using an oligonucleotide specific to the phoD gene (Fig. 2 and S3). They show that *Cobetia amphilecti* N-80 is not closely related to either *Cobetia amphilecti* B2M13 or *Cobetia amphilecti* KMM296 but is more closely related to the others. All of the above shows that *Cobetia amphilecti* N-80 still has some differences from the other strains in its phoD gene, although their 16S rRNA genes are extremely similar. To further determine the species identity of strain 80, we performed ANI analysis (Table 2) on the whole-genome sequences of the three strains. The results were more than 95%, which further confirmed that N-80 belonged to *Cobetia amphilecti.* Thus, we provided another complete genomes for *Cobetia amphilecti*.

There are many common genes in genus *Cobetia* (Fig. 4), including the functional genes of *acc*A, *acc*B, *acc*C, *acc*D, *fdh*A, *icd*, *ppc*, and *nir*D. It is possible that the genus *Cobetia* is generally able to fix inorganic carbon and plays an important role in deep-sea carbon cycle. The specific genes in *Cobetia amphilecti* N-80 are *lpt*E (LPS-assembly lipoprotein LptE), *drm*D(DISARM system SNF2-like helicase DrmD), *cad*R (Cd(II)/Pb(II)-responsive transcriptional regulator), *sod*X (nickel-type superoxide dismutase maturation protease), which may be the reason why *Cobetia amphilecti* N-80 can adapt to the deep-sea environment with low temperature, high pressure and high oxygen content. There are not many common genes in *Halomonas*, and it has certain functional diversity. For example, *Halomonas sulfidaeris* has *nap*B, *nap*C, *acc*A, and *icd*, and *Halomonas olivaria* has *nar*V, *nos*D, *nos*L, *nos*R, *nos*Y, and *acc*A. Although they are different, they are involved in the nitrogen metabolic pathway, and *Halomonas* genera should play a more important role in the deep sea nitrogen cycle.

### Comparison of the two strains

As two strains of different genera and different species, *Halomonas profundus* 13 and *Cobetia amphilecti* N-80 have great differences at the genetic level, but there are still many similarities. The 16S rRNA sequence similarity of *Halomonas marina* with any of the Halomonadaceae species was always below 95%, which is the limit generally accepted for genus delimitation. Therefore, they were isolated as a new genus called *Cobetia* in 2002 [1]; however, they are still highly similar to some other *Halomonas* species. It is also evident that *Halomonas profundus* 13 and *Cobetia amphilecti* N-80 share many genes, as shown in both the circos diagram (Fig. 3) and the Venn diagram (Fig. 5). There was also a high level of consistency in the genes involved in inorganic carbon fixation and nitrogen metabolism (Fig. 6). It can be seen from the COG (Fig. S4) and KEGG (Fig. S5) annotations [50] that most genes were greater in number in *Halomonas profundus* 13 than *Cobetia amphilecti* N-80, which may be because the larger genome of *Halomonas profundus* 13 contains more genes. From the difference between the two figures, the number of genes involved in essential metabolism (G in Fig. S4, carbohydrate metabolism and membrane transport in Fig. S5) and cellular activity (N in Fig. S4, cellular activity in Fig. S5) is far greater in *Halomonas profundus* 13 than in *Cobetia amphilecti* N-80.

At present, only one study related to *Halomonas profundus* is on polyhydroxyalkanoate production, and that study also clearly shows that the strain can use a wide variety of carbon sources [51]. However, there are many studies on nitrification and denitrification in the genus *Halomonas*. Four genes related to aerobic denitrification were reported in the genome of *Halomonas campisalis* ha3: *nap*A (encoding periplasmic nitrate reductase), *nir*S (encoding nitrite reductase), *nor*B (encoding nitric oxide reductase) and *nos*Z (encoding nitrous oxide reductase) [52], and the genome of *Halomonas* sp. strain B01 contains genes encoding ammonia monooxygenase (*amo*A) and nitrate reductase (*nar*H) [53]. The related genes that we found in *Halomonas profundus* 13 are *nar*G, *nar*Y, *nar*I, *nir*S, *nor*B, *nor*C, and *nos*Z, as well as some regulatory proteins related to nitrogen metabolism, *nar*T, *nar*X, *nar*L, *nir*Q, and *nir*D. Species within this genus can generally grow at concentrations of 1–12% NaCl, and the most salt-tolerant of these species is *Halomonas icarae* D1-1^ T^ [15], which can grow in 24% NaCl. The optimum NaCl concentration is generally 3–8%, with the highest optimum of 10% for *Halomonas pellis* L5^T^ [14–18, 54, 55]. Due to its halophily, this genus has good potential for application in nitrogen removal from wastewater. A novel moving-bed biofilm reactor constructed by inoculation with heterotrophic nitrifying–aerobic nitrifying bacteria, was proposed to dispose of high ammonia nitrogen wastewater [56], and species within this genus with nitrification and denitrification abilities can be applied to biotreatment of hypersaline wastewater [57]. *Halomonas profundus* 13 is a weak halophile, growing at NaCl concentrations ranging from 2–18% (optimum 8%, w/v), and can use more carbon sources such as glucose, arabinose, mannitol, maltose, gluconate, capric acid, adipic acid, malic acid, citric acid and phenylacetic acid according to the API 20NE. Therefore, it has a certain research value and application potential.

The previous studies of *Cobetia amphilecti* focused solely on *Cobetia amphilecti* KMM296 and reported on a novel alkaline phosphatase/phosphodiesterase [2], and its antibiofilm activity and biopreservative effect on meat products [58]. However, sequences for both *Cobetia amphilecti* B2M13 and *Cobetia amphilecti* KMM296 were found in NCBI. The GC contents of *Cobetia amphilecti* N-80, *Cobetia amphilecti* B2M13 and *Cobetia amphilecti* KMM296 are all 62.5%. However, the genome assembly lengths of *Cobetia amphilecti* N-80, *Cobetia amphilecti* B2M13 and *Cobetia amphilecti* KMM296 are 4.16 Mb, 4.29 Mb, and 3.97 Mb, with 1, 27, and 97 scaffolds, respectively. *Cobetia amphilecti* N-80 is more studied than *Halomonas profundus* 13 and has been shown to possess ammonia-N degrading [59], a novel glutaminase-free L-asparaginase [60], antibiofilm activity and biopreservative effects on meat products [61], bacteriocinogenic potential [62] and so on. This may be due to the wider distribution of *Cobetia amphilecti* N-80, which is found in seawater culture ponds [59], mangrove sediments [60], ready-to-cook meats [61], ecosystems within the Sea of Japan [62] and elsewhere. However, there are no detailed reports on its nitrification and denitrification abilities. We found that *nir*D can directly use NH_4_^+^ to generate energy and that there are additional related regulatory factors, such as *nar*T, *nar*X, *nar*L, and *nir*Q, in *Cobetia amphilecti* N-80. It is interesting that no complete denitrification pathway has been found in *Cobetia amphilecti* N-80 even though this species can survive in denitrification medium. We speculate that this may be associated with SO_4_^2−^ [63]. Through the gene function annotation information, we found that the genes related to assimilatory sulfate reduction in *Cobetia amphilecti* N-80 were *cys*NC, *cys*N, *cys*D, *cys*H, *cys*J, and *sir*, and the genes encoding sulfate/thiosulfate transport system proteins were *cys*A, *cys*W, and *cys*P. This suite of genes helps the microorganisms to transport extracellular SO_4_^−^ into the cell. The growth of *Cobetia amphilecti* N-80 in denitrification medium may be enabled by the assimilatory sulfate reduction pathway, which also mediates C, N and S circulation in the deep sea. Alternatively, this species may possess other new denitrification pathways that have not been found, or it may possess both sulfate reduction and novel denitrification pathways.

Deep-sea microorganisms can fix CO_2_ and other inorganic carbon in the deep sea to synthesize organic matter, provide energy for other organisms, and promote the storage of marine carbon pools. At the same time, biochemical processes such as synthesis and metabolism affect the coupling of the biogeochemical cycles of multiple elements in the deep sea. The ocean represents a major reservoir of nitrogen and sulfur on Earth. Both sulfur and nitrogen must be assimilated into organic metabolites [64, 65]. While nitrogen is mainly used for structural macromolecules, sulfur plays critical roles in the catalytic or electrochemical functions of biomolecules in cells [66]. *Cobetia amphilecti* N-80 may play a role in the C, N and S cycles of the ocean, which is worth further study.

## Conclusions

In this study, two strains with nitrification and denitrification abilities were isolated from the marine sediments obtained in the sea area near Antarctica. After identifying the species of the two strains, the whole genomes of the two strains were sequenced, and the basic characteristics of their genomes were determined. The complete genome sequence for *Cobetia amphilecti* was provided and filled a gap among *Halomonas profundus* genome sequences, which lays the foundation for further studies on these two species. *Halomonas profundus* 13 can utilize more carbon sources than *Cobetia amphilecti* N-80, as indicated by the API results as well as COG and KEGG prediction results. Finally, by analyzing the strains’ nitrification and denitrification abilities as well as their ability to fix inorganic carbon, the relevant metabolic pathways of both strains were predicted. We found 7 genes related to prokaryotic fixation of inorganic carbon and 5 genes related to nitrification–denitrification in *Cobetia amphilecti* N-80 and 8 genes related to prokaryotic fixation of inorganic carbon and 14 genes related to nitrification–denitrification in *Halomonas profundus* 13. Both *Halomonas profundus* 13 and *Cobetia amphilecti* N-80 could provide electrons and energy for their own fixation of inorganic carbon through their own nitrogen metabolism. This study provides molecular markers and theoretical support for the study of the C and N cycles involving microorganisms in the ocean and provides two new strains for carbon storage and utilization.

## Methods

### Isolation and screening of strains

The specific sampling locations for *Cobetia amphilecti* 80 and *Halomonas profundus* 13 were 39°48.890'W, 61°50.208'S, water depth 3389 m and 48°45.001'W, 60°10.219'S, water depth 1517 m, respectively. The culturable strains were obtained by using the methods of gradient dilution coating and streaking inoculation from the sediment samples on marine ZoBell 2216E medium (peptone 5 g; yeast extract 1 g; filtered seawater: ultrapure water (v/v) = 2: 1). Then, the bacteria were screened by inoculation in nitrifying medium ((NH_4_)_2_SO_4_ 1 g; CH_3_COONa 2.5 g; C_6_H_5_Na_3_O_7_ 2.5 g; K_2_HPO_4_ 0.2 g; MgSO_4_ 0.1 g; filtered seawater: ultrapure water (v/v) = 2: 1) and denitrifying medium (KNO_3_ 0.61 g; CH_3_COONa 2 g; K_2_HPO_4_ 0.2 g; MgSO_4_ 0.05 g; filtered seawater: ultrapure water (v/v) = 2: 1).

### DNA extraction and identification

A single colony purified on solid screening medium was inoculated into liquid 2216E medium and cultured at 15 °C for 1–2 days. To identify the strains, 16S rRNA gene amplicons were generated by PCR using primers 27F (5’-AGAGTTTGATCCTGGCTCAG-3’) and 1492R (5’-GGTTACCTTGTTACGACTT-3’). The template for 16S rRNA amplification was prepared by immersing 200 μl of bacterial culture solution in boiling water for 10 min and then immediately placing it in an ice box for 5 min. The PCR volume was 50 μL. The following conditions were used for the bacterial 16S rRNA gene amplification: initial denaturation at 94 °C, denaturation at 94 °C, annealing at 55 °C, elongation at 72 °C and a final extension step at 72 °C. The PCR products were sent to Sangon Biotech for sequencing. Similar sequences were obtained by NCBI BLAST of the 16S sequencing results, and a phylogenetic tree was constructed by MEGA-X, to determine the phylogenetic relationships of each strain. Multiple sequence alignments were conducted by ClustalW in MEGA X with default parameters. The alignment result was then used to construct a phylogenetic tree based on the neighbor-joining (NJ) method of MEGA X, with the following setups: Maximum Composite Likelihood and pairwise deletion. Maximum likelihood trees were estimated using MEGA X and the best DNA/Protein model were selected by MODELS in MEGA X. The Tamura 3-parameter model T92 + G + I was used to produce the Maximum likelihood tree of 16S rRNA, and the Jones-Taylor-Thornton model JTT was used to produce the Maximum likelihood tree of PhoD. Both NJ and ML trees were conducted 1000 bootstrap replications. API 20NE (BioMérieux, France) was used to study the physiological and biochemical patterns of the strains, which serves as an identification system for nonfastidious, nonenteric gram-negative rods. Average nucleotide identity (ANI) analysis was conducted on JSpeciesWS (http://jspecies.ribohost.com/jspeciesws/#analyse).

### Whole genome sequencing

First, the two strains were enriched and cultured in marine ZoBell 2216E medium and centrifuged to remove the supernatant. Then, the precipitated bacteria were frozen in liquid nitrogen for five minutes and sent to Wuhan Onemore-tech Co., Ltd. for Illumina and Nanopore sequencing. The genome assembly uses Software Unicycler (0.4.9). First scaning the size of the k-mer thoroughly via SPAdes (v3.14.1) to ensure that there is no linear looped sequences. Subsequently the greedy algorithm is used to generate the illumina stitching sketch which is determined by the copy number of overlapping. By simplifying the repetitive sequence between single copy contigs into one sequence, the map was simplified. Then use Bowtie2 ( 2.4.1) and Pilon to refine the sequence, use accurate short reads to correct contig, and then use long reads to assembly. Using BWA (0.7.17-r1198-dirty) software, the Illumina short sequence reads were aligned to the assembled genome to obtain the second-generation sequencing depth statistics. The third-generation sequencing depth statistics were generated by using Minimap 2 (2.17-r974-dirty) to align the long sequences to the assembled genome. We used the Samtools (1.10–71–gb298f29) depth tool to assist with the calculation of the average sequencing depth. A 2,000 bp sliding window was used to determine the read coverage in different regions. Prokka (1.13) software was used to predict the coding genes within the assembled genome. Prodigal (v2.6.3) was used to predict the coding genes, Aragorn (v1.2.38) was used to predict tRNAs, RNAmmer (1.2) was used to predict rRNAs, and Infernal (1.1) was used to predict miscRNAs. The predicted gene elements were summarized, and preliminary annotation was completed. Pseudofinder was then used to detect the pseudogene candidate sequences in annotated GenBank files from bacterial and archaea genomes.

### Bioinformatics analysis

To obtain comprehensive gene function information, we annotated the gene functions according to the following eight databases: UniProt, RefSeq, Pfam, NR, TIGERFAMs, GO, KEGG [50], COG and KEGG Pathway [50]. The predicted gene sequences were compared with COG, KEGG, Swiss-Prot, RefSeq and other functional databases by BLAST + (2.9.0 +) to obtain the gene function annotation results. Gene functions were annotated using HMMER software (3.3.1) based on the Pfam and TIGERFAM databases. The genome circle map was plotted using the R package circlize. Visualization of the genome through these analyses is conducive to exploring and clearly evaluating the relationships among genome components and locations. The relationship between the genomes of the two strains was obtained by BLAST analysis on linux with EValue less than10^−5^. When E value is less than 10^–5^, it indicates that the two sequences have high homology rather than calculation error. The results of synteny with the sequencing length and density of the two strains were visualized using the module of Graphics in TBtools to plot the comparative genomic circos map. The genome and annotation information of the same genus were obtained from NCBI, and the Venn diagram was drawn by Jvenn [67]. Genes related to nitrification and denitrification or to inorganic carbon fixation were found in the KEGG [49] (https://www.kegg.jp/) and GO (http://geneontology.org/) databases. These genes were plotted in a Venn diagram along with the *Halomonas profundus* 13 and *Cobetia amphilecti* N-80 gene annotation results to obtain their correlations. Finally, the metabolic pathway maps of the two strains were predicted according to the obtained nitrification and denitrification genes and inorganic carbon fixation-related genes.

## Supplementary Information

**Additional file 1:** **Figure S1.** The Tamura 3-parameter model T92+G+I was used to produce theMaximum likelihood tree of 16S rRNA.

**Additional file 2:** **Figure S2.** Circular genome atlas of the two strains. Different colorsrepresent different results, including GC content, GC skew, the depth ofSequencing, CDS, rRNA and tRNA.

**Additional file 3:** **Figure S3.** The Jones-Taylor-Thornton model JTT was used to produce theMaximum likelihood tree of PhoD.

**Additional file 4:** **Figure S4.** COG functional classification of genes.

**Additional file 5:** **Figure S5.** KEGG databaseannotation [1].

**Additional file 6:** **Figure S6.** Sequencealignment of the protein translated by a functional gene with other homologousproteins. The information of the proteins used was downloaded from NCBI, withthe abbreviations in front such as *Cobetia amphilecti* as Ca and *Halomonasprofundus* as Hp, followed by accession in parentheses.

**Additional file 7:** **Table S1.** The comparison data of the two genomes.

**Additional file 8:** **Table S2**. The location of the functional gene in the genome of *Cobetiaamphilecti* N-80.

**Additional file 9:** **Table S3.** The locationof the functional gene in the genome of the *Halomonas profundus* 13.

## Data Availability

The datasets analysed during the current study are available in the NCBI repository, accession numbers: NC_CP084115 for *Cobetia amphilecti* N-80, complete genome; CP086344 for *Halomonas profundus* 13, complete genome.
