# Supplementary figures and images for: Complete genome sequencing and comparison of two nitrogen-metabolizing bacteria isolated from Antarctic deep-sea sediment
Source: BMC Genomics. 2022 Oct 19;23:713. doi: 10.1186/s12864-022-08942-6 (PMC9580203; doi:10.1186/s12864-022-08942-6)

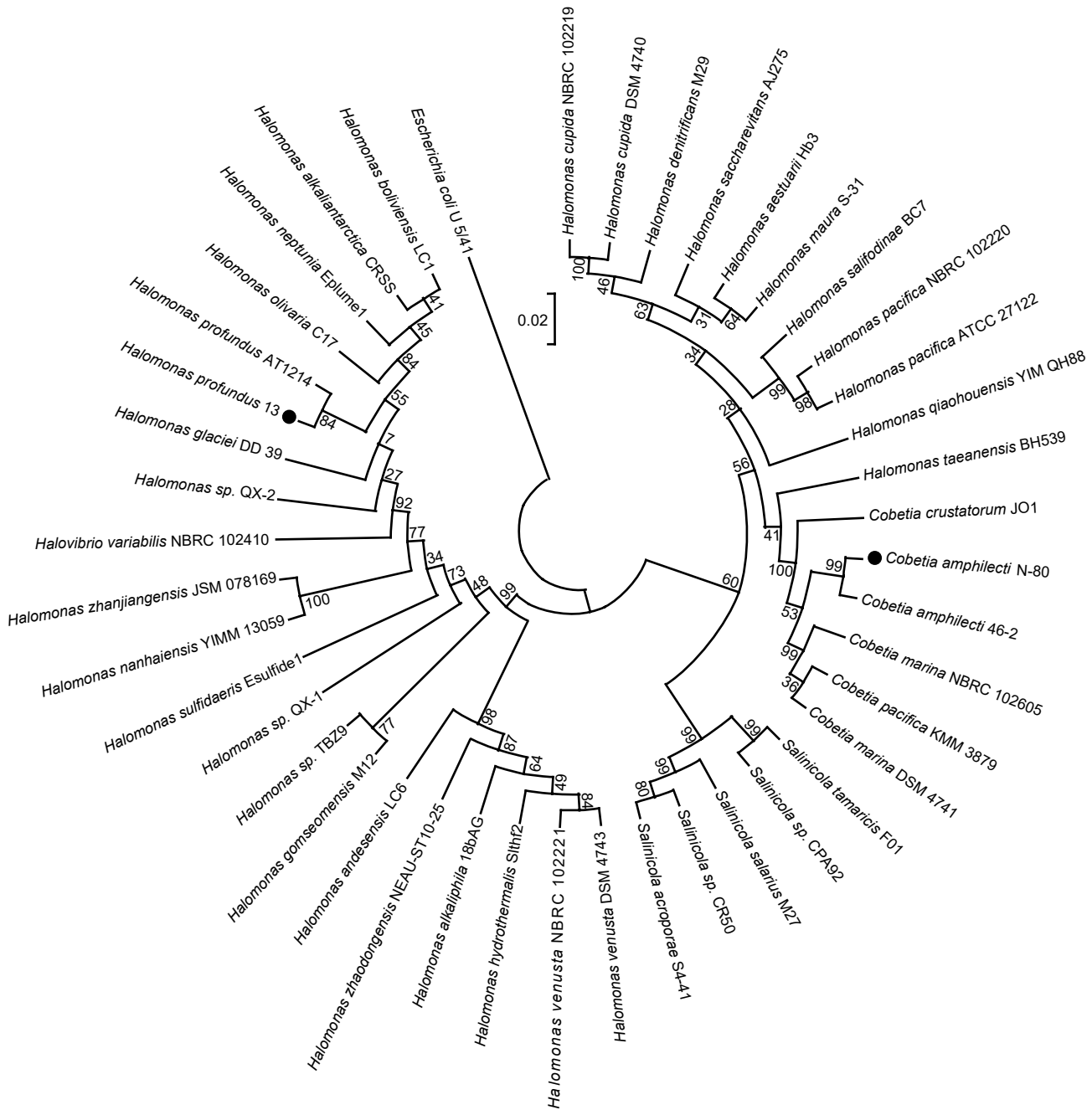

Supplement: Supplementary file 1 — Additional file 1: Figure S1. The Tamura 3-parameter model T92+G+I was used to produce theMaximum likelihood tree of 16S rRNA. [file 12864_2022_8942_MOESM1_ESM.pdf]

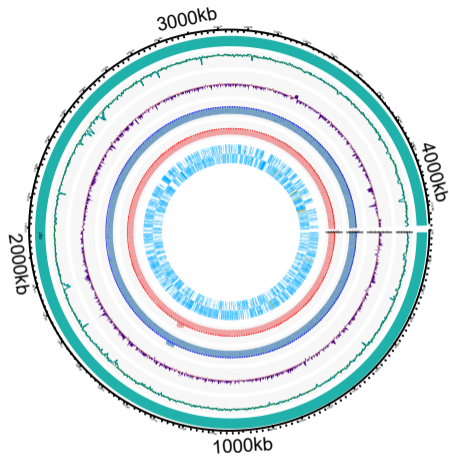

a: *Cobetia amphilecti* N-80

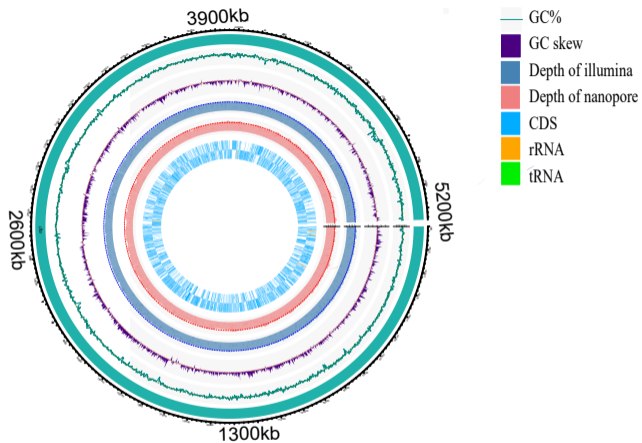

b: *Halomonas profundus* 13

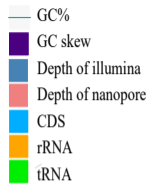

Supplement: Supplementary file 2 — Additional file 2: Figure S2. Circular genome atlas of the two strains. Different colorsrepresent different results, including GC content, GC skew, the depth ofSequencing, CDS, rRNA and tRNA. [file 12864_2022_8942_MOESM2_ESM.pdf]

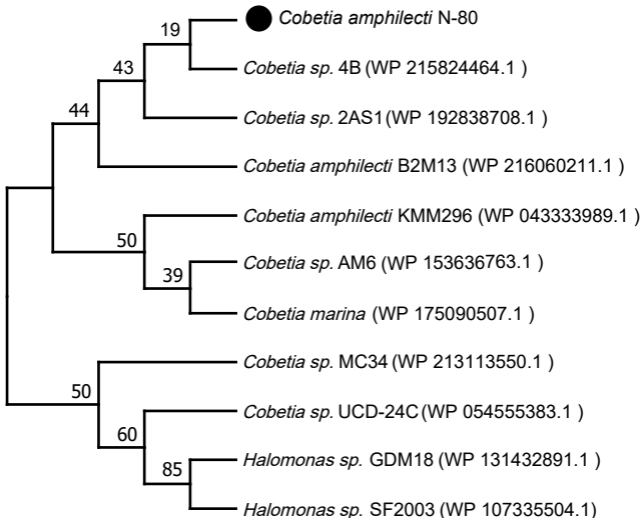

Supplement: Supplementary file 3 — Additional file 3: Figure S3. The Jones-Taylor-Thornton model JTT was used to produce theMaximum likelihood tree of PhoD. [file 12864_2022_8942_MOESM3_ESM.pdf]

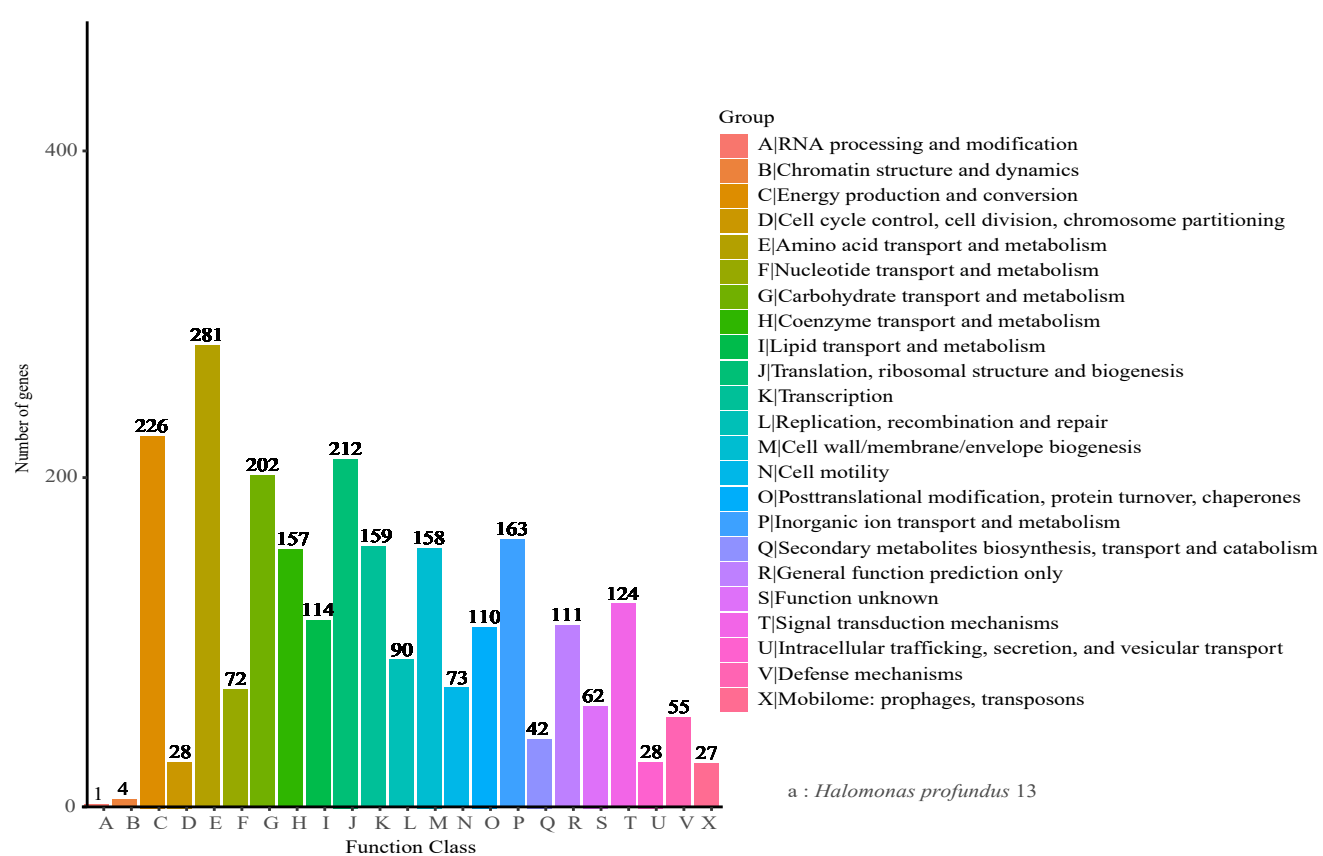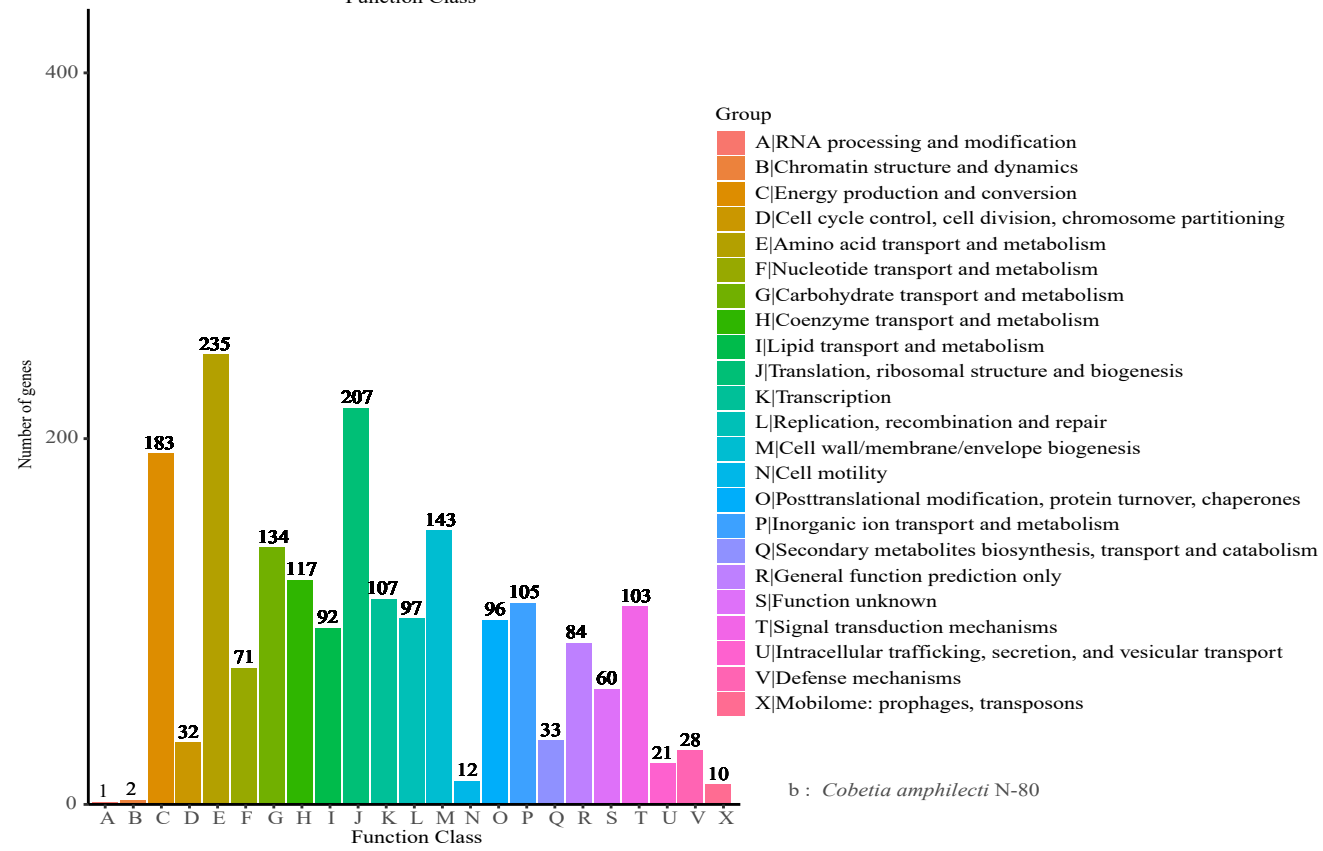

Supplement: Supplementary file 4 — Additional file 4: Figure S4. COG functional classification of genes. [file 12864_2022_8942_MOESM4_ESM.pdf]

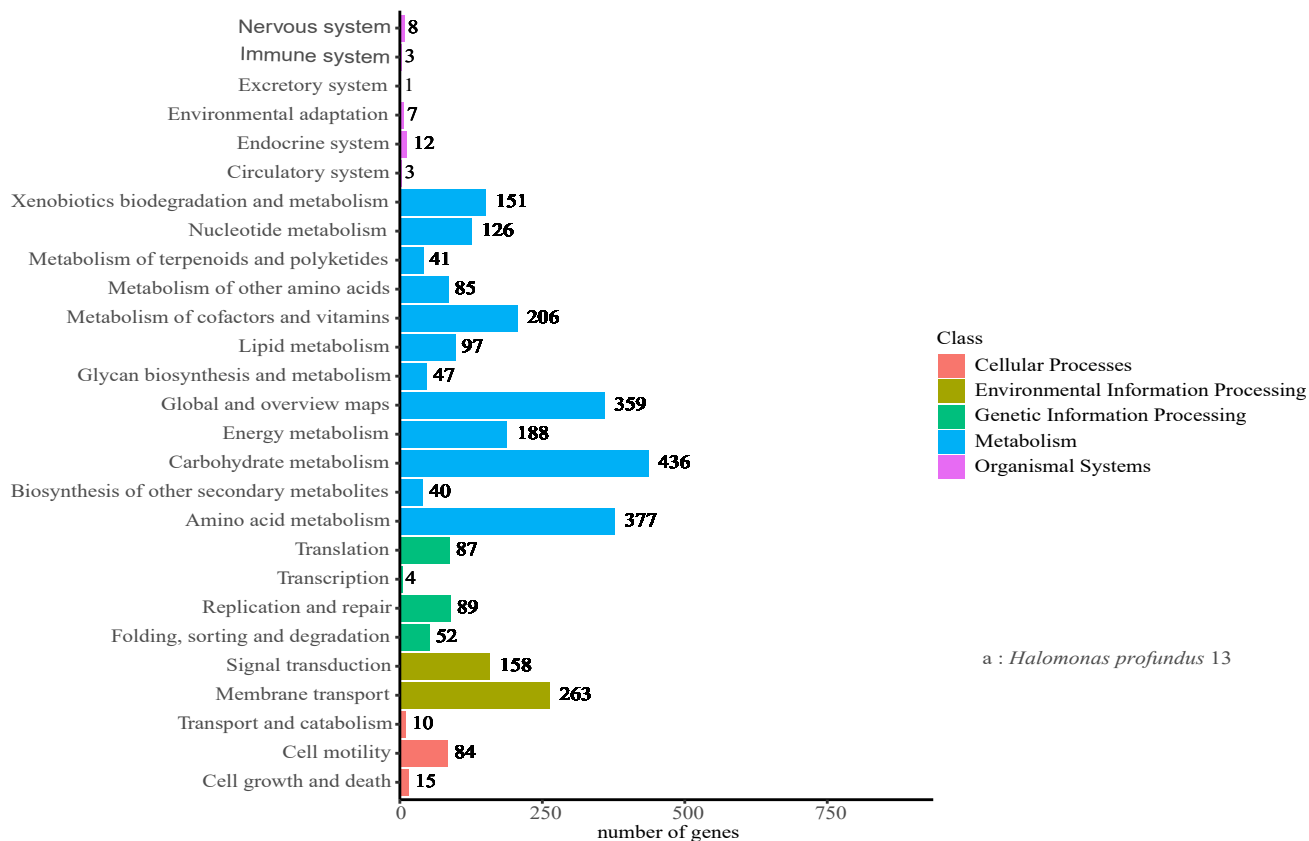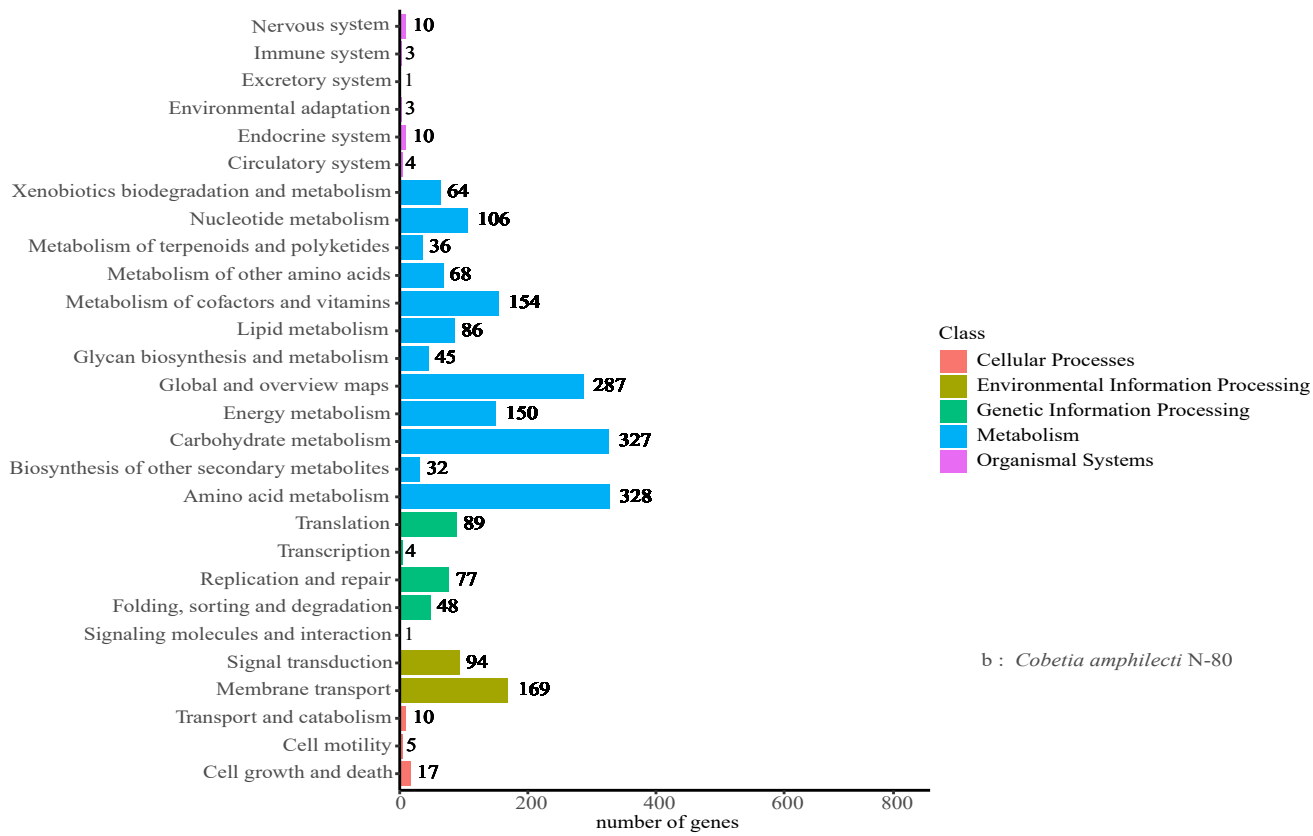

Supplement: Supplementary file 5 — Additional file 5: Figure S5. KEGG databaseannotation [1]. [file 12864_2022_8942_MOESM5_ESM.pdf]
