# Supplementary material for: Complete genome sequencing and comparison of two nitrogen-metabolizing bacteria isolated from Antarctic deep-sea sediment
Source: BMC Genomics. 2022 Oct 19;23:713. doi: 10.1186/s12864-022-08942-6 (PMC9580203; doi:10.1186/s12864-022-08942-6)
Supplement: Supplementary file 6 — Additional file 6: Figure S6. Sequencealignment of the protein translated by a functional gene with other homologousproteins. The information of the proteins used was downloaded from NCBI, withthe abbreviations in front such as Cobetia amphilecti as Ca and Halomonasprofundus as Hp, followed by accession in parentheses. [file 12864_2022_8942_MOESM6_ESM.pdf]

acsA

```
HpacsA                    -------------------------MTATSQSLPDYYHYLDNFSVDRVLAR--LDDHRDGL---LNAFEACCGRHVRAGRGDVLAL   56
CaacsA                    MTPSDTTTNRTSRAASAASANSHAMQPAASQTLSDYLAAFDIQALRDELFTTPLDTNDEGE--LGNAYEACVGRHLRAGQGERIAL   84
CcacsA(WP_144726521.1)    MTTRDTTTNRTSRAANVTSATDATFEPTPHQSLQEYLASFDIDALRSELLASGLESEPEPERFTGNAFEACVGRHLRAGRGESIAL   86
HkacsA(WP_181514112.1)    ---------------------------MSTQSLPGYSAFMERFSIDEVIAR--LDDHADGK---FNAYESCCGRHLRAGRGEVPAL   54
HvacsA(WP_106230398.1)    ---------------------------MST--LPDYQSYLETFSIDDVIAR--LDDHRDGK---ANAFESSCGRHLRAGRGDVLAL   52

HpacsA                    VQEDSQGDMHTLSYAELDEQSAKLAGWFKSQGLGIGDRIACMLPRSPQLLIAVLATWRIGAVYQPLFTAFGPDAVDYRLGRAGTKL   142
CaacsA                    VHEDEQGRVRELSYAQLDEQSARMAAALKAQGVMAGQRVACMLSRTPELMIALAATWRLGAVYQPLFTAFGTDALDYRLSRAETQI   170
CcacsA(WP_144726521.1)    VHEDEQGNVSEMSYAALDDLSARMATALAAQGVVPGDRVACMLSRTPELLVALAATWRLGAVYQPLFTAFGPDALEYRLARAETRV   172
HkacsA(WP_181514112.1)    IHEDAQGHVSRLTYAELETESAKLAGWFAERGLGVGDRIACMLPRSPQLLVAVLATWRIGAVYQPLFTAFGPDAVDYRLGRADTKL   140
HvacsA(WP_106230398.1)    VHEDTQGQVNTLTYAELDAESARLAGWFAERGLGVGDRIACMLPRSPQLLVAVLATWRIGAVYQPLFTAFGPDAVDYRLGRADTRL   138

HpacsA                    VITDHANRYKFDGLSQCPPVLAVGGA-TSEHADDHDWQAALTHTSMSDKPPRLAPEAPFLQMFTSGTVGKPKGVAVPLAGMTAFAI   227
CaacsA                    VITETSQRSKFTELARHPVIICVRGEGDALEADDLDWQTLMQSEPLTEPPVWLPAEAPFLQMFTSGTVGKPKGVAVALNALPAFWL   256
CcacsA(WP_144726521.1)    VITETAQRFKFNELERHPSIICVRRRDESLQDGDLDWQVLVQSEPLVQPPVPLSSDAPFLQMFTSGTVGKPKGVAVALNALPAFWL   258
HkacsA(WP_181514112.1)    VITDHDNRFKFDGLSQCPPVLAVGGP-SEGHGDDLDWSEALSHSPIEANPPRLSPELPFLQMFTSGTVGKPKGVAVPLAGMPAFAI   225
HvacsA(WP_106230398.1)    VITDHANRFKFDGLTQCPPVLAVGGP-DADHADDLDWNNALAHAPIETTPPRLSREEPFLQMFTSGTVGKPKGVAVPLAAMPAFAL   223

HpacsA                    YMELAIDLRDDDRFWNMADPGWAYGLYYAIAGPLLLGVTTHFCEAGFSAEGALAFMKRHHITNFAAAPTAYRLMKASGLFDDAHET   313
CaacsA                    YQRLAVDLRPGERFWNMADPGWAYGLYYAITGPLLLGATTYFMQAPFSAEGGLAFMERHRIDNFAAAPTAYRMLKASGLGEGAFER   342
CcacsA(WP_144726521.1)    YQRLAIDLRPDERFWNMADPGWAYGLYYAITGPLLLGATTYFMQSPFTAEGGLAFMERHRIDNFAAAPTAYRMLKASGVCEGAFER   344
HkacsA(WP_181514112.1)    YMELAIDLRESDRFWNMADPGWAYGLYYAIAGPLLLGVTTHFCEAGFSAEGALAFMQRHRITNFAAAPTAYRLMKASGLFDDAHEK   311
HvacsA(WP_106230398.1)    YMELAVDLRDTDRFWNMADPGWAYGLYYAIAGPLLLGVTTHFCEAGFSAEGALAFMKRHRITNFAAAPTAYRLMKASGLFDDAHQT   309

HpacsA                    LELRAASSAGEPLNTEVVTWVEKSLGCPVMDHYGQTETGMTCNNHHALDHLKHVGAMGVPMPGYRLAILDVEYKELPAGEPGVLAV   399
CaacsA                    LNLRVASSAGEPLNTEVVGWVARELGCAVMDHYGQTETGMTCCNHHALAHEVVVGSVGFPLPGYRLAVLDAEYRELPPGEPGVLAV   428
CcacsA(WP_144726521.1)    LNLRVASSAGEPLNTEVVGWVERELGCTVMDHYGQTETGMTCCNHHALAHEIVVGSVGFPLPGYRLAVLDAEYRELPPGEPGVLAV   430
HkacsA(WP_181514112.1)    LDLRVASSAGEPLNTEVVTWVERSLGCPVMDHYGQTETGMTCCNHHALEHPKHVGAMGMPMPGYRLAILDAEYNELPPGEPGVLAV   397
HvacsA(WP_106230398.1)    LELRAASSAGEPLNTEVVTWVERSLGCPVMDHYGQTETGMTCNNHHALDHPKHVGAMGVPMPGYRLAILDAEYRELPPGEPGVLAV   395

HpacsA                    DIKNSPAHFFQGYTWQEKNPFVEGYYLTGDVVIRNEDGTFQFAGRDDDIITTAGYRVGPTDVENSVMTHPAVAESAAVGQPDEIRG   485
CaacsA                    DIARSPAHFFAGYTWQEKQPCVEGYYLTGDVVVAEPDGRFTFAGRDDDIITTAGYRVGPADVENAVLVHPAVAESAAVGKPDALRG   514
CcacsA(WP_144726521.1)    DIARSPAHFFAGYTWQEKQPCVEGYYLTGDVVVAEPDGRFTFAGRDDDIITTAGYRVGPADVENAILAHPTVAESAAVGKPDDIRG   516
HkacsA(WP_181514112.1)    DIERSPAHFFQGYTWQEKHPFAKGYYLTGDVVIRNEDGTFQFAGRDDDIITTAGYRVGPTDVENTVLTHPAVAESAAVGQPDEIRG   483
HvacsA(WP_106230398.1)    DIENSPAHFFAGYTWQEKHPFAEGYYLTGDVVIRNEDGTFQFAGRDDDIITTAGYRVGPTDVENSVMTHPAVAESAAVGQPDEIRG   481

HpacsA                    EVIKSYIVLREGYEASDELADEIKQRVRERLSTHAFPRIIEFVDELPKTPSGKIQRFKLRAQAVEEAKASQ--   556
CaacsA                    EIIKAYVVLRDGHVGSDELAEEIRASVRQRLSAHSYPREIDFVDSLPKTPSGKIQRFLLRAQARAS-------   580
CcacsA(WP_144726521.1)    EIIKAYVVLRDGHVGSETLADEIRQSVRQRLSGHSYPREVAFVDSLPKTPSGKIQRFLLRAEARED-------   582
HkacsA(WP_181514112.1)    EIIKSYVVLREGFEASDALADEIRQQVRERLSTHAFPRVIEFVDTLPKTPSGKIQRFKLRADAAEKAETGAAQ   556
HvacsA(WP_106230398.1)    EIIKSYVVLREGYEASDALADEIRKQVRERLSTHAFPRVIEFVEELPKTPSGKIQRFKLRADAVDKAEEAPAK   554
```

fdhA

```
HpfdhA                  MN-AANRGVVYKGPGEVAVESIAYPELALGN----------RKCDHGVILKVVTTNICGSDQHMVRGRTTAPSGLVLGHEITGLVVEC  77
CafdhA                  MSANANRGVVYQGAGKVSVESLDYPTLALGN----------RKCEHGVILKVVSTNICGSDQHMVRGRTTAPQGLVLGHEITGLVVEA  78
PafdhA(ABG39378.1)      MNKTSNRGVVYKGPGQVAIESIAFPELAIGN----------RKCQHGVILEVVTTNICGSDQHMVRGRTTAPSGLVLGHEITGRVIEV  78
NkfdhA(WP_017574614.1)  --MPGNRGVVYQGPGEVDVTDIAYPEFELKDGPGVHPDNVGRKVPHGVIVEVVATNICGSDQHMVRGRTTAPQGLVLGHEITGKVVET  86
SsfdhA(WP_020606316.1)  --MCHNHGVAYISPGVVEVQEIEYPKLALGD----------RKCEHGVILQIVSTNICGSDQHMVRGRTTAPAGLVLGHEITGLVIEA  76

HpfdhA                  GRDVEFIQPGDLVSVPFNIACGRCRNCKEGRTGICLNVNPARPGAAYGYVDMGGWVGGQTEYVMVPYADFNLLKFPDADQAMEKIKDL 165
CafdhA                  GRDVEFIKVGDIVSVPFNIACGRCRNCKEGKTGICLNVNPARPGAAYGYVDMGGWTGGQTDYVMVPYADFNLLKFPDADQAMEKIRDL 166
PafdhA(ABG39378.1)      GRDVEFIKPGDLVSVPFNIACGRCRNCKEGRTGICLNVNPARPGAAYGYVDMGGWVGGQTEYVMVPYADFNLLKFPDPEQAIEKIQDL 166
NkfdhA(WP_017574614.1)  GPDVEFVQEGDLVSVPFNISCGRCRNCKARRTEICLNVNPEKPGSAYGYVGMGGWVGGQAQYALVPYADWNLLVFPDRDQAMDKIMDL 174
SsfdhA(WP_020606316.1)  GRDVEFIKEGDLVSVPFNIACGRCRNCKVGQTGICLNVNPARPGAAYGYVDMGGWVGGQAEYVMVPYADFNLLKFPDKDQAMAKIRDL 164

HpfdhA                  TLLSDIFPTGFHGCVTAGVGPGSTVYIAGAGPVGLAAVSAQLLGAACVIVGDMVEERLAQARSFGCETIDLTQDGDMADKIEVILGE 253
CafdhA                  TLLSDIFPTGFHGCVTAGVGPGSTVYIAGAGPVGLAAVSAQLLGAACVIVGDMIPERLQQAESFGCETIDLRQEGSMEDKVEVILGE 254
PafdhA(ABG39378.1)      TLLSDIFPTGFHGCVTAGVGPGSTVYIAGAGPVGLAAASAQLLGAACVIVGDMIEERLAQARSFGCETIDLTQEGDMGDRIEAIIGE 254
NkfdhA(WP_017574614.1)  TMLSDIFPTGYHGCVTAGVGVGSSVYIAGAGPVGLAAAAAQLLGAAVVIVGDLKEQRLAQARVFGCETVNVS-EGDPADLVEGVLGT 261
SsfdhA(WP_020606316.1)  TLLSDIFPTGYHGAVTAGVGPGSIVYVAGAGPVGLACAVSCHLLGAAVVIVGDMIPERLEQARSFGCETVDLRKDTPLADQIAEIIGV 252

HpfdhA                  REVDAFVDCVGFEAHACGCNHGKEAPATVLNSAMSLTRAGGQIGIPGLYVTEDPGAADDAAKQGALSMRFGLGWAKSHSFHTGQCPVM 341
CafdhA                  REVDCFVDCVGFEAHGCGCDHGREQPATVLNSAMALTRAGGQIGIPGLYVTEDPGAEDEAAKVGALSMRFGLGWAKSHSLHTGQCPVM 342
PafdhA(ABG39378.1)      REVDAFVDCVGFEAHACGHNHHQESPATVLNSAMKMTRAGGQIGIPGLYVTEDPGAVDDAAKQGALSMRFGLGWAKSHSFHTGQCPVM 342
NkfdhA(WP_017574614.1)  REVDSAVDAVGFEAYGTGEEAGSEAPASVLNTAMDVTRAGGSIGIPGLYVTGDPGASDEAAKEGSLSIRFGLGWSKSHAFFTGQCPAM 349
SsfdhA(WP_020606316.1)  PEVDSAVDCVGFEAHGHGSDSGKEQPATVLNAAMTVTRAGGAIGIPGLYVTGDPGASTEDAKEGNLSIRIGLGWAKSHSFYTGQCPVM 340

HpfdhA                  KYHRPLMQAILFGKVNIADAVNVQMITLDQAPQGYADFDGGAAKKFVIDPHGSVA----- 396
CafdhA                  KYHRPLMQAILFGRVNIADAVNVQVISLEEAPQGYADFDGGAAKKFVIDPHAMVRNGRAA 402
PafdhA(ABG39378.1)      KYHRALMQSILFDKVKIAKAVNVKMISLDDAPKGYADFDGGAAMKFVIDPHGNAA----- 397
NkfdhA(WP_017574614.1)  KYHRELMMAILHEKVHIADAVNAVALPLEEAARGYQEFDRGAAKKYVLDPNGYLGTG--- 406
SsfdhA(WP_020606316.1)  KYHRQLMNAILYDKVQIAKAVNVEVISLDKAPKGYQDFDKGAAKKFVIDPHGLIPN---- 396
```

icd of *Halomonas profundus* 13

```
Hpicd                    MTKTPKIIYTLTDEAPALATHSLLPIIDAFTDSAGIQVETRDISLAARIIAQFPDYLSEEQRIGDHLAELGELTKTPEANIIKLPNISA  89
Alicd(WP_148254375.1)    MSKTPKIIYTLTDEAPALATHSLLPIIDAFTDPAGIEVETRDISLAGRIIALFPDYLSEAQRLGDHLAELGDLAKTPEANIIKLPNISA  89
Mticd(WP_224420928.1)    MSKSPKIIYTLTDEAPALATHSLLPIIDAFTDPAGIDVETRDISLAGRIISLFPEYLTDEQRIADDLAELGELTKTPEANIIKLPNISA  89
Hsicd(WP_163502434.1)    MSKTPKIIYTLTDEAPALATHSLLPIIDAFTDPAGIEVETRDISLAGRILSQFPDYLSDEQQLGDHLAELGELAKTPEANIIKLPNISA  89
Mticd(WP_224415265.1)    MSKSPKIIYTLTDEAPALATHSLLPIIDAFTDPAGIDVETRDISLAGRIISLFPDYLSEEQRIADDLAELGELTKAPEANIIKLPNISA  89

Hpicd                    SLPQLKAVIKELQQQGYKLPDYPEAPQSDEEKDIQARYDKTKGSAVNPVLREGNSDRRAPLSVKNYARKYPHRMGEWSSDSKSHVAHMS 178
Alicd(WP_148254375.1)    SIPQLKAAIKELQQQGYKLPNYPEEPQNAEEQEIKSRYDKVKGSAVNPVLREGNSDRRAPQSVKSYVRKYPHRLGEWSADSRSHVAHMQ 178
Mticd(WP_224420928.1)    SVPQLKAAIKELQSQGYKLPDYPEEPGNDTERDIKARYDRVKGSAVNPVLREGNSDRRAPQSVKNFARKYPHRLGEWSADSRSHVAHMS 178
Hsicd(WP_163502434.1)    STPQLKAAIRELQQQGYKLPNYPDEPSSQVEHDVKTRYDRTKGSAVNPVLREGNSDRRAPQSVKGFARKYPHRMGEWSADSKSHVASMS 178
Mticd(WP_224415265.1)    SVPQLKAAIKELQSQGYKLPDYPEDPRDDTERDIKARYDRVKGSAVNPVLREGNSDRRAPQSVKNFARKYPHRLGEWSADSRSHVSHMS 178

Hpicd                    EGDFYGSEKSALIEKATDLKIELVAKDGSTTVLKQKTSVLEGEVVDAAVMSRNALRRFVADEVADAKAKDVLFSLHLKATMMKVSDPIM 267
Alicd(WP_148254375.1)    EGDFYSSEKSAMIATAGNLKIELVADDGSTQTLKETTPVLAGEVVDAAVMSNRKLRRFVAAEIEDAKSRNVLFSLHLKATMMKVSDPII 267
Mticd(WP_224420928.1)    DGDFYGSETSAVMESAGSVKIALHGADGSVKVLKEAVPVLAGEVIDAAVMSRQALRRFVAEAIEDAKSQDVLFSLHLKATMMKVSDPIM 267
Hsicd(WP_163502434.1)    DGDFYASEKSALIEAAGNVKIELVAADGSTQVLKEKTPVLAGEVIDGSVMSRRVLSAFISEQIEDAKQQDVLFSLHLKATMMKVSDPII 267
Mticd(WP_224415265.1)    DGDFYASETSAVMAAEGSVKVTLHGDDGNVTVLKEAVPVLAGEVIDAAVMSSQALRRFVAEAIEDAKSQDVLFSLHLKATMMKVSDPIM 267

Hpicd                    FGMVVSEFYQDVLSKHADDLQQAGFDPNNGIGDLYSAIKSLPEERQTQIEDEIQALYAERPRLAMVNSHKGITNLHVPSDVIIDASMPA 356
Alicd(WP_148254375.1)    FGAVVSEFYREVLDKHAGELDKVGFDPNNGIGDLYSTIKALPEAKQNEITSDIESLYAQRPPLAMVNSHKGITNLHVPSDVIIDASMPA 356
Mticd(WP_224420928.1)    FGIVVSEFYKEVLAKYADDLKQVGFDPNNGIGDLYATIQSLAEDKRQAIESDIQALYAERPSLAMVNSHKGITNLHVPSDVIIDASMPA 356
Hsicd(WP_163502434.1)    FGVAVEAFYRDVLARHAADLDKAGFNANSGIGDLYATIKELPEAKQEAIKADVEALYAERPRLAMVNSHKGITNLHVPSDVIVDASMPA 356
Mticd(WP_224415265.1)    FGIVVSEFYKEVLAKYADDLKQVGFDPNNGIGDLYATIQSLAEDKRQAIESDIQALYAERPSLAMVNSHKGITNLHVPSDVIIDASMPA 356

Hpicd                    MIRDSGKMWGADDQLHDVKAVIPDRCYAGIYQTVIDDCKQHGAFDPTTMGSVPNVGLMAQKAEEYGSHDKTFQIPTDGTVRVTDTSGNV 445
Alicd(WP_148254375.1)    MLRDSGQMWGADDALHDTKAVIPDRCYAGIYQTVIEDCRQNGAFDPTTMGSVPNVGLMAQKAEEYGSHDKTFQIPADGSVRVTDGQGNV 445
Mticd(WP_224420928.1)    MIRDSGKMWGADDALHDTKAVIPDRCYAGIYQVVIEDCKKNGAFDPTTMGSVPNVGLMAQKAEEYGSHDKTFHIPAAGTVRVTDAQDNV 445
Hsicd(WP_163502434.1)    MIRDSGKMWGADDQLHDAKAVIPDRCYASIYQTVIDDCKANGAFDPTTMGSVPNVGLMAQKAEEYGSHDKTFEIPADGTVRVSDESGAL 445
Mticd(WP_224415265.1)    MIRDSGKMWGADDALHDTKAVIPDRCYAGIYQVAIEDCKENGALDPTTMGSVPNVGLMAQKAEEYGSHDKTFQIPAAGTVRVTDAQGNV 445

Hpicd                    VLEHSVEQGDIWRMCQTKDAPVRDWVKLAVNRARESGTPAIFWLDAQRAHDAQLIKKVESYLKEHDTTGLDIRIMTPEEAMRFSLERIR 534
Alicd(WP_148254375.1)    IFEHGVETGDIWRMCQTKDAPIQDWVKLAVTRARASNTPAVFWLDANRAHDAQLIQKVERYLKDHDTAGLDIRIMAPEDAMRFSLERIR 534
Mticd(WP_224420928.1)    VFEHRVEEGDIWRMCQTKDAPIQDWVKLAVARARDSQTPAVFWLDANRGHDAQLIEKVERYLKDHDTDGLDIRIMTPEDAMRFSLERIR 534
Hsicd(WP_163502434.1)    LFEHAVERGDIWRMCQTKDAPIQDWVKLAVTRARDSGAPAIFWLDAKRAHDAQLISKVETYLEDYDTSGLDIRILAPVEAMQVSLERIR 534
Mticd(WP_224415265.1)    VFEHAVEEGDIWRMCQTKDAPIQDWVKLAVARARDSQTPAVFWLDANRGHDAQLIEKVERYLKDHDTDGLDIRIMTPEDAMRFSLERIR 534

Hpicd                    KGEDTISVTGNVLRDYLTDLFPIMELGTSAKMLSIVPLMNGGGLFETGAGGSAPKHVQQLLEENHLRWDSLGEFLALAASLEHLGSTFD 623
Alicd(WP_148254375.1)    QGQDTISVTGNVLRDYLTDLFPIMELGTSAKMLSIVPLMNGGGLFETGAGGSAPKHVQQLLEENHLRWDSLGEFLALAASLEHLGESFG 623
Mticd(WP_224420928.1)    AGKDTISVTGNVLRDYLTDLFPIMELGTSAKMLSIVPLMNGGGLFETGAGGSAPKHVQQFLEENHLRWDSLGEFLALAASLEHLGKTFD 623
Hsicd(WP_163502434.1)    KGEDTISVTGNVLRDYLTDLFPIMELGTSAKMLSIVPLMNGGGLFETGAGGSAPKHVQQFLEENHLRWDSLGEFLALAASLEHLGNAFG 623
Mticd(WP_224415265.1)    AGKDTISVTGNVLRDYLTDLFPIMELGTSAKMLSIVPLMNGGGLFETGAGGSAPKHVQQFLEENHLRWDSLGEFLALAASLEHLGKTFD 623

Hpicd                    NARAKMLAKALDQANGKFLDSNKSPSRKVGELDNRGSHFYLALYWAEALAEQSEDSEMQTLFGRLADALTAKEATIVDELNSVQGQPAD 712
Alicd(WP_148254375.1)    NQRAKLLADALDQANGKFLDSDKSPKRKVGELDNRGSHFYLALYWAQALAEQDGDSDLKALFARLAETLEANEATIVNELAKVQGQPVD 712
Mticd(WP_224420928.1)    NARARVLAKALDQANGEFLDSNKSPSRKVGEIDNRGSHFYLALYWAKAVAAQNDDAELKAQFAELARKLEENEDAILAELNGVQGQPVD 712
Hsicd(WP_163502434.1)    NVRATLLAKALDSANGKFLDSNKSPSRKVGELDNRGSHFYLAMFWAEALAAQDEDAELKALFARLAESLKSNEAAILNELNGVQGKPVD 712
Mticd(WP_224415265.1)    NARARVLAKALDQANGEFLDSNKSPSRKVGEIDNRGSHFYLALYWAKAVAAQDDDAELKAQFAELARKLEENEDAILAELNGVQGQPVD 712

Hpicd                    IGGYFHPDHELTAKVMRPSQTLNETLAMVAKG 744
Alicd(WP_148254375.1)    IRGYYHPDFDLTRQAMRPSRTFNDALAMVAKG 744
Mticd(WP_224420928.1)    IRGYYHPDYALTSQAMRPSKTLNDALGVVKD- 743
Hsicd(WP_163502434.1)    IRGYYHPDHDLTREAMRPSRTLNEALNVVANG 744
Mticd(WP_224415265.1)    IRGYYHPDHALTSQAMRPSKTLNDALGVVKD- 743
```

icd of *Cobetia amphilecti* N-80

```
Caicd                   MGFQKIVVPEGGAKITANSDNTLNVPNNPIIPFIEGDGIGVDVTPAMKIAIDAAVDKAYGGERKIHWMEVYAGEKATHVYDADTWLP   87
Psicd(WP_064482494.1)   MGYQKIQVPASGDKITVNTDNTLNVPNNPIIPYIEGDGIGVDISPVMIKVVDAAVEKAYGGERKIAWMEIYAGEKATQVYDQDTWLP   87
Pzicd(WP_128121199.1)   MGYQKIQVPASGDKITVNTDNTLNVPNNPIIPYIEGDGIGVDISPVMIKVVDAAVEKAYGGERKIAWMEIYAGEKATQVYDQDTWLP   87
Haicd(WP_027351550.1)   MAFEKIQLPAGGEKITVNADNSLNVPNEPIIPFIEGDGIGVDVTPAMKIAVDAAVAKAYGGERKIHWMEVYAGEKATTIYDADTWLP   87
Pbicd(PPC77059.1)       MGYQKIQVPENGQKITVNADMSLNVPNNPIIPFIEGDGIGVDVSPVMIKVVDAAVEKAYGGERKIAWMEVYAGEKATQVYDENTWLP   87

Caicd                   EETLEAVKDYIVSIKGPLTTPVGGGIRSLNVALRQKLDLYTCLRPVRWFTGVPSPVKKPADVDMVIFRENSEDIYAGVEWKAGTPEA  174
Psicd(WP_064482494.1)   KETLEVVRDFVVSIKGPLTTPVGGGIRSLNVALRQELDLYVCQRPVRWFTGVPSPVKKPADVDMVIFRENSEDIYAGVEWKAGSPEC  174
Pzicd(WP_128121199.1)   KETLEVVRDFVVSIKGPLTTPVGGGIRSLNVALRQELDLYVCQRPVRWFTGVPSPVKKPADVDMVIFRENSEDIYAGVEWKAGSPEC  174
Haicd(WP_027351550.1)   EETLRAVQEYVVSIKGPLTTPVSGGIRSLNVALRQKLDLYVCERPVRWFKGVPTPVKKPQDVDMVIFRENSEDIYAGVEWKAGSAEA  174
Pbicd(PPC77059.1)       AETLEVVKDYVVSIKGPLTTPVGGGIRSLNVALRQELDLYVCQRPVRWFTGVPSPVKNPADVEMVIFRENSEDIYAGVEWKAGSPEA  174

Caicd                   DKVIKFLREEMGVTNIRFPENCGIGVKPVSEEGTKRLVRQAVQYTIDNDRESLTLVHKGNIMKFTEGAFKDWGYEIVKDEFGGELLD  261
Psicd(WP_064482494.1)   EKVIKFLTEEMGVKKIRFTENCGIGIKPVSLEGTKRLVRKALQYAVDNDRRSVTIVHKGNIMKFTEGAFKEWGYEVARDEFGAELLD  261
Pzicd(WP_128121199.1)   EKVIKFLTEEMGVKKIRFTENCGIGIKPVSLEGTKRLVRKALQYAVDNDRRSVTIVHKGNIMKFTEGAFKEWGYEVARDEFGAELLD  261
Haicd(WP_027351550.1)   DKVIEFLTKEMGVTNIRFTDECGIGVKPVSKEGTQRLVRKAIQYAIDNDRESVTLVHKGNIMKFTEGAFKNWGYEVAVEAFGAEPLD  261
Pbicd(PPC77059.1)       EKVIKFLTEEMGVKKIRFTEGCGIGIKPVSEQGTKRLVRKALQYTIDNDRSSLTLVHKGNIMKFTEGAFKEWGYEVARDEFGAELLD  261

Caicd                   GGPWMTFKNPNNGKDIVVKDVIADAMLQQILLRPAEYDVIATLNLNGDYLSDALAAEVGGIGIAPGANLSDTVAMFEATHGTAPKYA  348
Psicd(WP_064482494.1)   GGPWMQFKNPNTGKNIVVKDAIADAMLQQILLRPAEYDVIATLNLNGDYLSDALAAEVGGIGIAPGANLSDTIAMFEATHGTAPKYA  348
Pzicd(WP_128121199.1)   GGPWMQFKNPNTGKNIVVKDAIADAMLQQILLRPAEYDVIATLNLNGDYLSDALAAEVGGIGIAPGANLSDTVAMFEATHGTAPKYA  348
Haicd(WP_027351550.1)   GGPWHAFDNPNTGKRIVIKDVIADAMLQQVLLRPEEYDVIATLNLNGDYLSDALAAQVGGIGIAPGANRSDAVGMFEATHGTAPKYA  348
Pbicd(PPC77059.1)       GGPWMTMKNPRTGKQIVIKDVIADAMLQQILLRPAEYDVIATLNLNGDYLSDALAAEVGGIGIAPGANLSDDVAMFEATHGTAPKYA  348

Caicd                   GQDKVNPGSVILSAEMMLRHMGWVEAADLVLKGVEKAIAKGEVTYDFHRLMDDAKLLKCSEFGQAVADNMDA  420
Psicd(WP_064482494.1)   GQDKVNPGSLILSAEMMLRHMGWVEAADLIIKSTESAIAAKTVTYDFERLMDGAKLMSCSEFGDAMISHM--  418
Pzicd(WP_128121199.1)   GQDKVNPGSLILSAEMMLRHMGWVEAADLIIKSTESAIAAKTVTYDFERLMDGAKLMSCSEFGDAMISHM--  418
Haicd(WP_027351550.1)   GQDKVNPGSLILSAEMMLRYMGWTEAADLILQGVENAIAKGEVTYDFHRVMENAKLLKCSEFGEAVAANM--  418
Pbicd(PPC77059.1)       GQDKVNPGSVILSAEMMLRHMGWTEAADLIIKGTEGAIAAKTVTYDFERLMEGAKLLKCSEFGDAIVANM--  418
```

narG

```
HpnarG                    ---MSHFIDRLNFFRKSREPFANEHGELRSESRQWEDSYRARWQHDKVVRSTHGVNCTGSCSWKIYVKNGLVTWETQQTDYPRTRPD   84
AanarG(WP_019699762.1)    ---MSHFLDRLTYFSQPRESFAGGHGQVNGEDRTWEDAYRNRWAHDKTVRSTHGVNCTGSCSWKIYVKGGIVTWETQQTDYPRTRWD   84
AxnarG(WP_006386096.1)    ---MSHFLDRLKFMSRVKSTYADGHGEVVNEDRKWENAYRARWQHDKVVRSTHGVNCTGSCSWKVYVKNGLITWETQQTDYPRTRPD   84
BtnarG(WP_010677859.1)    MAKQNSLFRSLKHLVR-GERINDGWTEENPRPRDWESIYRQRWAHDKIVRSTHGVNCTGSCSWKIHVKDGIIAYETQQTDYPSTGDN   86
EhnarG(WP_006810897.1)    ---MSKFLDRFRYFKQKGETFADGHGQVLDTNRDWEDGYRQRWQHDKVVRSTHGVNCTGSCSWKIFVKNGLVTWEMQQTDYPRTRPD   84

HpnarG                    LPNHEPRGCPRGASYSWYLYSANRLKYPLIRKPLLALWREALKANPDPVDAWASIVEDPAKTKQYKRARGMGGFVRGNWDELNELVA  171
AanarG(WP_019699762.1)    MPNHEPRGCARGASYSWYLYSANRVKYPMVRGRLLKAWRAARAT-MGPVEAWAAIVGDEATRQGWQRVRGLGGFARSSWDEVNEIVA  170
AxnarG(WP_006386096.1)    LPNHEPRGCPRGASYSWYVYSAQRVKYPMMRGRLMEMWREARKT-MDPIAAWEWISQDPARARRYKSVRGLGGFVRTDWDTATEMIA  170
BtnarG(WP_010677859.1)    FPDYEPRGCPRGASFSWYTYSPTRVKYPYVRGDLLALWKEELANADNPVQAWENIVTDPEKRKSYVSARGKGGFVRGNWKDVCQMIA  173
EhnarG(WP_006810897.1)    MPNHEPRGCPRGASYSWYLYSANRLKYPLMRKRLMKMWREAKVQHSDPVDAWASIIEDADKAKSFKQARGRGGFVRSSWKEVNELIA  171

HpnarG                    ASNVYTAKQYGPDRILGFSPIPAMSMVSYAAGSRYLSLIGGVCMSFYDWYCDLPPASPMTWGEQTDVPESADWYNSGYIIAWGSNVP  258
AanarG(WP_019699762.1)    AANIHTIRRHGPDRIIGFSPIPAMSMVSYAAGSRYLSLIGGVCMSFYDWYCDLPPSSPQVWGEQTDVPESADWYNAGYIIAWGSNVP  257
AxnarG(WP_006386096.1)    AANAYTIKKFGPDRLIGFSPIPAMSMVSYAAGARYLSLLGGACLSFYDWYCDLPPASPQIWGEQTDVPESADWYNSTYLMVWGSNVP  257
BtnarG(WP_010677859.1)    ASTIYTIKKYGPDRVVGFSPIPAMSMVSYAGGARFLSLIGGTILSFYDWYADLPPASPQVWGDQTDVPESGDWYNTKYFIIWGTNIP  260
EhnarG(WP_006810897.1)    ASNVYTVKTYGPDRVAGFSPIPAMSMVSYASGARYLSLIGGTCLSFYDWYCDLPPASPQTWGEQTDVPESADWYNSSYIIAWGSNVP  258

HpnarG                    QTRTPDAHFFTEVRYKGTKTVSITPDYAEVSKLTDEWLSAKQGTDAALAMAMGHVILKEFHLDN---PSAYFTDYVRRYTDMPFLVE  342
AanarG(WP_019699762.1)    QTRTPDAHFFTEVRYKGTKTVAVTPDYSEVAKLADLWMNPKQGTDAAVAMAMGHVILKEFYFPEQGERSAYFDEYVRRYTDMPMLVT  344
AxnarG(WP_006386096.1)    QTRTPDAHFYTEVRYKGTKTVAVSSDFGEMVKFGDIWLAPKQGTDAALAMAMGHVILKEFHLSG---ASAYFRDYVRRYTDMPMLVL  341
BtnarG(WP_010677859.1)    QTRTPDAHFMVESRYNGTKVVGVSPDYAEYDKFADIWLPAKAGTDGALAMAMTHVILKEFYVDK---ETPYFRDYAKQYTDLPFLIT  344
EhnarG(WP_006810897.1)    QTRTPDAHFFTEVRYKGTKTVAVTPDYAEIAKLCDLWLAPKQGTDAAMALAMGHVMLREFHLDK---PSQYFTDYVRRYTDMPMLVM  342

HpnarG                    LEVRE----DGSFAPGRQLRASDFDAALGQENNPEWKTVAWDETRDQLVVPRGSIGFRWGETG----DEVGKWNLESLD-AAGTEIK  420
AanarG(WP_019699762.1)    LREQALPDGRVVRVPDRYVRASDFADRLGQGNHPEWKTVALDAH-GEVVCPQGAIGFRWGP---DGRADQGQWNLEDKDARTGDAVR  427
AxnarG(WP_006386096.1)    LKER---DG--FYAPDHFLRASQLDGALGEQNNPDWKTLVFDETSGDLVAPNGSIGFRWGEGAVNGGEKVGRWNLEARDGGSGRAIE  423
BtnarG(WP_010677859.1)    LNKKG-----ENYRSDRFLRASDFTDQHELG---EWKTVVWDENTNQIAVPNGSQGFRWDSS--------TKWNLDMTT-EDGTSIN  414
EhnarG(WP_006810897.1)    LEER-----DGYYAAGRMLRAADLVDALGQENNPEWKTVACNSN-GELVAPNGSIGFRWGEK--------GKWNLEQRNGTTGEETE  415

HpnarG                    PLLSLANH-HDSVAR-----VAFPYFGGIEHEHFEHVKGAGVGAADDLLFHNLPAKRLKRADGS----EMLAVTVFDLMCANYGIDR  497
AanarG(WP_019699762.1)    LRLSLMEAGADEAHGWETAQVGFPYFGGIASEHF------PANVQSDVIARTVPVRRLATVDAGGTAREVLVATVFDLQVAQYGVAR  508
AxnarG(WP_006386096.1)    PRLSLVDG-ADAVVG-----VGFPYFG---------------AEHDEVLVRNVPVRRIRLADGS----EALAATVYDLQMANYGLDR  485
BtnarG(WP_010677859.1)    PKLSFIKE-SDETVM-----VEFPYFAE---------------KEGGKVVRGVPVKRMKDNSGN----ELMVTTVYDLMLAHTGINR  476
EhnarG(WP_006810897.1)    LRLSMLGS-QDEIAD-----VGFPYFGGEGSEHFNKVE------LQNVLMHKLPVKRLQLADGS----TALVTTVYDLTMANYGLER  486

HpnarG                    GFVGDGEDDGATSFDQIRPYTPAWQEKITGVPAEQCTRIAREFADNAHKTNGRSMIIVGAGMNHWYHMDMNYRGLINMLVMCGCIGQ  584
AanarG(WP_019699762.1)    GLP---GENAAAGYDDDTPYTPAWQEKITGTPRAQVIAVARQFAENAHRTRGRSMVIIGAAMNHWYHCDMNYRGIINMLMMCGCIGQ  592
AxnarG(WP_006386096.1)    GLG---GGNVAASYDDDVPYTPAWQEKHTGVPRAQVIQVAREFAQNAHDTEGKSMVIVGAGLNHWYHMDMIYRGIINMLMMCGCVGK  569
BtnarG(WP_010677859.1)    GLK---GDYPTDYNDAARPYTPAWQESITGVKKEHVIQVAREFAENAALTQGKSMIAMGGGTNHWFHSDQIYRSILNLVLLTGSQGV  560
EhnarG(WP_006810897.1)    GLN---DENCATSYDDVKAYTPAWAEQITGVPRAQITRIAREFGENADKTHGRSMIIVGAGLNHWYHLDMNYRGLINMLIFCGCVGQ  570

HpnarG                    SGGGWAHYVGQEKLRPQTGWTPLAFGLDWQRPPRHMNSTSFFYSHSSQWRYEKLEIKEILSPLAKAEDYPG-SLIDFNVRAERMGWL  670
AanarG(WP_019699762.1)    SGGGWAHYVGQEKLRPQTGWTALAFALDWVRPPRQQNSTSFFYAHTDQWRYEKLGVEEVLSPLADRAQFGG-SMIDYNVRAERMGWL  678
AxnarG(WP_006386096.1)    SGGGWAHYVGQEKLRPQFGWAPLAFASDWVRPPRQMNGTSFFYAHTSQWRHEKLNVQEVLGPTADRGRYGDLSMIDLNARAERMGWL  656
BtnarG(WP_010677859.1)    NGGGWAHYVGQEKVRPLEGFSQIAFANDWVKAPRLMNGTSFFYFATEQFRYE-YDFDDRETDWG--SKYSNMHPADFNAMSARLGWL  644
EhnarG(WP_006810897.1)    SGGGWAHYVGQEKLRPQTGWQPLAFALDWQRPARHMNSTSYFYNHSSQWRYETVTAQELLSPMADKSRYSG-HLIDFNVRAERMGWL  656

HpnarG                    PSAPQLDTNPLRLAKKAEAAGMST---ADYAVQQLKSGELRFAAEDPDAPENFPRNMFIWRSNLLGSSGKGHEYMLKYLLGTRHGIQ  754
AanarG(WP_019699762.1)    PSAPQLQTNPLQVVRDAEAAGLDP---KAYAVQGLQSGALKMSCTDPDHPDNWPRNMFVWRSNILGSSGKGHEYFLKHLLGTRNGVQ  762
AxnarG(WP_006386096.1)    PSAPQLRTNPLDLTAQAEAAGKDP---VAHAVEQLKSGELRMSCEDPDDPANFPRNMFVWRSNILGSSGKGHEYFLKYLLGTQNAVF  740
BtnarG(WP_010677859.1)    PSFPTLSQNSLDVLKEARSRNSDDKAVIDDIAKQLVDGKLDFAIENPNDPRNFPRVFFNWRSNLLGDSGKGHEYFVKHLIGSKDTVL  731
EhnarG(WP_006810897.1)    PSAPQLGTNPLRIAEAAKQAGMSP---VDYTVKSLKDGSIRFAAEQPENGKNHPRNLFIWRSNLLGSSGKGHEYMLKYLLGTENGIQ  740

HpnarG                    GKDLGDFGGQKPEEVVWRDEAPEGKLDLLVTLDFRMSTTCLYSDIVLPTATWYEKDDLNTSDMHPFIHPLTAATDPAWESRSDWDIY  841
AanarG(WP_019699762.1)    GKDLGE-GDARPAEVEWHAQAPEGKLDLLVTLDFRMSTTCLYSDIVLPTATWYEKNDLNTSDMHPFIHPLSTAVDPAWQSRSDWEIY  848
AxnarG(WP_006386096.1)    GDEAD---AIKPVEVNYQEDAAEGKLDLLTVLDFRMSTTCLYGDIVLPTATWYEKDDLNTSDMHPFIHPLSEAVQPLWESKTDWEIY  824
BtnarG(WP_010677859.1)    SDPEQ---SWQPKDVNISEKPPEGKTDLFISMDFRMTSSGLFSDIILPAATWYEKQDISTTDLHPFVHPFNAAISPPWETRSDWDAF  815
EhnarG(WP_006810897.1)    GKDLGKQGGVKPEEVEWKDNGLDGKLDLVVTLDFRLSSTCLYSDIVLPTATWYEKDDMNTSDMHPFIHPLSAAVDPAWESKSDWEIY  827

HpnarG                    KGIAKAFSKVCVGHLGEETDLVTLPMQHDSPGELAQP--AVMDWKKGECAPIPGKTMPSLIEVKRNYPETYERFTSVGPLLESIGNG  926
AanarG(WP_019699762.1)    KGFARAFSALAPGHLGVERDLVLSPIAHDTPGELAQPF-GVADWTRGECDLIPGKTGPQMSVVERDYPNLYRRFTALGPLMEKVGNG  934
AxnarG(WP_006386096.1)    KLLARKFSDIGGPYLGKRRDLVLTPLMHDTPGELGQAF-EPKDWKLGECDLVPGKTAPNMTVVERDYNDIYRKFTSVGPLLDKLGNG  910
BtnarG(WP_010677859.1)    REIAKAFSELAKDHLPAQEDLVISPLAHDTISEIAQPFGKVKDWRKGEVEGIPGKTMPNFNFVNRDYPNVYDMWITVGPNIKN-GYG  901
EhnarG(WP_006810897.1)    KDIAKKFSEVCVGHLGKETDVVTLPIQHDSAAELAQPL-DVKDWKKGECDLIPGVTAPHIIPVERDYPATYERFTSIGPLMEKIGNG  913

HpnarG                    GKGIAWNTDAEVELLGKLNHRKLD-GPHKGRPLIDSAIDAAEMILTLAPETNGAVAVKAWDALSKITGRDHTHLARPKFDEKIRFRD 1012
AanarG(WP_019699762.1)    GKGIGWDTKDEVQQLRELNGTVPGNGPTQGMPRIDSDIDACEVILQLAPETNGHVAVKAWDALGRQTGRDHTHLALHREDEKIRFRD 1021
AxnarG(WP_006386096.1)    GKGINWNTEHEVGELAGINERVDEPGVSQGRPRLDTAIDAAEMILTFAPETNGHVSVKAWEALGKITGRDHTHLAVGREHDKIRFRD  997
BtnarG(WP_010677859.1)    TKGVKIPGDKVFESLMDRLGPSKSVGVGKGLPSLYSDVKAINAILLMSGATNGKRAVEGWKSMEQKTGKKLRSISEAREEEDFTLDA  988
EhnarG(WP_006810897.1)    GKGIAWNTQSEMDLLRKLNYTKAD-GPAKGQPMLNTAIDAAEMILTLAPETNGHVAVKAWAALSEFTGRDHTHLAKNKEEEKIRFRD  999

HpnarG                    VVAQPRKIISSPTWSGLEDEHVSYNAGYTNVHELIPWRTVSGRQQFYQDHAWMRAFGESLLVYRPPIDTKAAKGFQLPADNGFKSKA 1099
AanarG(WP_019699762.1)    VQAQPRKIISSPTWSGLESEKVSYNAGYTNVHELIPWRTLTGRQQFYIDHPWMQAFGEGFSSYRPPVDLKATAEVQGRKPNGNPEIA 1108
AxnarG(WP_006386096.1)    IQAQPRKIISAPTWSGLESEEVSYNAGYTNVHELIPWRTLTGRQQFYQDHRWMLDFGEGSCVYKPAIDTKTVAPMLGRYPNGEKELV 1084
BtnarG(WP_010677859.1)    LTIQPRQTLSTPVWSGLESDGRRYSPFTVNKEYHIPWHTLTGRQSFYLDHEVMLDYGEGLPLYLPPINKGPFIKGEKEVENSGKSIT 1075
EhnarG(WP_006810897.1)    IQAQPRKIISSPTWSGLEDEHVSYNAGYTNVHELIPWRTLSGRQSLYQDHQWMRDFGESLLVYRPPIDTRSVKAVMGAKSNGNPEKA 1086
```

```
HpnarG                    LNFLTPHQKWGIHSTYSDNLLMLTLNRGGPVVWLSEADAAEIDIEDNDWIEVYNANGSIAARAVVSQRVKAGMVMMYHAQERNVNVP 1186
AanarG(WP_019699762.1)    LNFITPHQKWGIHSTYTDNLLMLTLSRGGPCVWISEDDAKRAQIEDNDWIELFNANGALTARAVVSQRVKPGMVMMYHAQEKIVNTP 1195
AxnarG(WP_006386096.1)    LNWITPHQKWGIHSTYSDNLRMLTLSRGGPHVWISEAEARQAGLVDNDWVEVFNVNGTLTARVVVSQRVPVGMCLMYHAQEKIVNVP 1171
BtnarG(WP_010677859.1)    LRYMTPHQKWGIHTMFTDTRNMVQLFRGWQVVWLNEEDAEIGIKDNDWIELYNRNGAVVARAVLTYRMPRGAVYMHHAQDRTMGVP 1162
EhnarG(WP_006810897.1)    LNFLTPHQKWGIHSTYSDNLLMLTLSRGGPIVWMSEADAKDLGIEDNDWIEVFNSNGALTARAVVSQRVPAGMTMMYHAQERIVNLP 1173

HpnarG                    GSEVTGTRGGIHNSVTRVCPKPTHMIGGYAQLSYSFNYYGTVGSNRDEFVLVRKMKHVDWLDGE--GNDSVQKNSAQEAVK 1265
AanarG(WP_019699762.1)    GSEVTGTRGGIHNSVTRVVLKPTHMIGGYAQLSYGFNYYGTIGTNRDEFIVLRKMNRVDWLDTP-VADPLVRPTLDQGETA 1275
AxnarG(WP_006386096.1)    GAETSGKRGGIHNSVTRTVLKPTHMIGGYAQQAYGFNYYGTVGANRDEFVVLRKMKKVDWLEGPLVEDPAVQTQQEEKQS- 1251
BtnarG(WP_010677859.1)    GNTINKNRGGTHNSVTRIYPKATHMIGGYSQLSYGFNYYGPTGSQRDTLTIVRPLKEVDWLEN------------------ 1225
EhnarG(WP_006810897.1)    GSEITEQRGGIHNSVTRITPKPTHMIGGYAQLAYGFNYYGTVGSNRDEFVVVRKMKNINWLDGE--GNDQVQ-----ESVK 1247
```

narI

```
HpnarI                 MYDAIAHYLTHLIYGYYPYLAGTVFLLGSLLRFDHGQYTWKTGSSQMLSSKNMRLGSNLFHIGIIVIFFGHLVGMLTPHWVYEPFLHA  88
PbnarI(WP_090438698.1) ---MFFEYLQHLIYGYYPYLAGTVFLVGSLLRFDHGQYTWKAHSSQILSKKNMRLASNLFHVGILVIFFGHLFGMLTPHWVYAPFISA  85
PbnarI(WP_090500693.1) ---MFFEYLQHLIYGYYPYLAGTVFLVGSLLRFDHGQYTWKAHSSQILAKKNMRLASNLFHVGILVIFFGHLFGMLTPHWVYAPFISA  85
PanarI(WP_147185795.1) ---MFGEYLQHLIYGYYPYLAGTVFLVGSLLRFDHGQYTWKTGSSQMLSTKHMRLASNLFHIGILVIFFGHLFGMLTPHWVYAPFLTA  85
AlnarI(WP_148252143.1) ---MFAEYLQHLIYGYYPYLAGTVFLVGSLLRFDHGQYTWKTGSSQMLSSKHMRLASNLFHIGIIVIFFGHLFGMLTPHWVYAPFLSP  85

HpnarI                 GTKQLVAIVVGGIAGAMCVVGGAMLLYRRLANPRVKASSSMMDTLILGLIVLQACLGMVTIIFSLGHMDGEMMLTLSSWAQSIVFFSG 176
PbnarI(WP_090438698.1) GNKQLLAIIIGGIAGAMCIVGGAMLLYRRMTNPRVKASSATMDNVILALILFQACLGMITIIFSLGHLEGDVMLTLASWAQAIVFFSG 173
PbnarI(WP_090500693.1) GNKQLLAIIIGGLAGAMCIIGGAMLLYRRLTNPRVKASSATMDNLILALILFQACLGMITIIFSLGHLEGDVMLTLASWAQAIVFFSG 173
PanarI(WP_147185795.1) GTKQILAIVIGGIAGVMVLIGGGMLLYRRLYNPRVKASSSFMDTLILGILVVQAALGIITVFFSLGHLDGEMMLTLAGWAQSIVFFSG 173
AlnarI(WP_148252143.1) GAKQILAIVIGGIAGVLCVVGGAMLLYRRLFNPRVRASSGFMDTLILAILVFQAGLGLVTVFFSLGHLDGEMMLTLASWAQAIVFFGG 173

HpnarI                 GAADYMQEVSWIYKLHIFIGLTIILLFPFSRLVHVWSAPFGYVTRRYQLVRRRG 230
PbnarI(WP_090438698.1) GAANYLEEVSWIYKIHIFVGLTIILLFPFSRLVHVWSIPLGYLGRNYQIVRKRG 227
PbnarI(WP_090500693.1) GAANYLEEVSWIYKVHIFVGLTIILLFPFSRLVHIWSIPLGYLGRNYQIVRKRG 227
PanarI(WP_147185795.1) NAADYMANVSWIYKLHVFLGLTIILLFPFTRLVHVWSIPLGYLGRRYQIVRRRG 227
AlnarI(WP_148252143.1) GAADYMTEVSWIYKLHIFLGLTIILLFPFTRLVHVWSAPLGYVTRRYQLVRKRG 227
```

narY

```
HpnarY                       MKIRSQVGMVLNLDKCIGCHTCSVTCKNVWTSREGMEYAWFNNVETKPGIGYPKEWENQAKWKGGWMRRNDGRIEPRIGGKWRVLANI   88
SsnarY(WP_013172229.1)       MRIKAQIGMVMNLDKCIGCHTCSITCKNTWTNRQGAEYMYFNNVETKPGIGYPKQWEDQDRYKGGWERR-KGKLELKSGAKATRLLNL   87
AanarY(WP_008166145.1)       MRIRAQIGMVLNLDKCIGCHTCSVTCKNVWTSRDGVEYAWFNNVETKPGIGYPKEWENQGKWNGGWKRTAAGKLEPRQGGKLRILANL   88
EcnarY(WP_000702569.1)       MKIRSQVGMVLNLDKCIGCHTCSVTCKNVWTGREGMEYAWFNNVETKPGIGYPKNWEDQEEWQGGWVRDVNGKIRPRLGSKMGVITKI   88
LjnarY(WP_010530758.1)       MKIKAQVAMVMHLDKCIGCHTCSVTCKNTWTNRPGTEYMWFNNVETRPGTGYPKRWEDTERFKGGWTLK-NGKLQLKAGGPISKLMNI   87

HpnarY                       FANPDLPEMDDYYEPFTFDYQHLHTAKIGEHQPVARPRSLISGQRMKKIEWGPNWEEILGTEFAKRRKDANFDKVQADIYGQFENTFM  176
SsnarY(WP_013172229.1)       FYNPYQPSIEDYFEPWSYDYENLFDARERKTQPVARAKSQLTQDFIDLN-WGPNWEDDLAGAHITGLQDPNVKKMEESIRTEFEDVFM  174
AanarY(WP_008166145.1)       FANPNLPAIDDYYEPFTYDYEHLQNAPLSQTPPTARPVSVLTGKKMDKIEWGPNWEDDLGGEFSARSRDALFEGVQKEMYSTFENTFM  176
EcnarY(WP_000702569.1)       FANPVVPQIDDYYEPFTFDYEHLHSAPEGKHIPTARPRSLIDGKRMDKVIWGPNWEELLGGEFEKRARDRNFEAMQKEMYGQFENTFM  176
LjnarY(WP_010530758.1)       FYNPDMATMDDYYEPWTYNYEHLINSPKSENLPVARPQSVITGEYIDKPEWGSNWDDDLAGGTEVVSQDPTVEKLQDHISMEYEKTFM  175

HpnarY                       MYLPRLCEHCLNPTCVAACPSGAIYKREEDGIVLIDQDKCRGWRMCISGCPYKKIYYNWKSGKSEKCIFCYPRIEAGQPTICSETCVG  264
SsnarY(WP_013172229.1)       MYLPRICEHCINAPCVASCPSGAIYKRDEDGIVLVDQEACRGWRHCITSCPYKKVYFNWETNKAEKCTMCFPRIENGQPTICSETCVG  262
AanarY(WP_008166145.1)       MYLPRLCEHCLNPACVASCPSGSIYKREDDGIVLVDQDKCRGWRMCISGCPYKKIYYNWNSGKAEKCTFCYPRIEAGQPTVCSETCVG  264
EcnarY(WP_000702569.1)       MYLPRLCEHCLNPSCVATCPSGAIYKREEDGIVLIDQDKCRGWRLCISGCPYKKIYFNWKSGKSEKCIFCYPRIESGQPTVCSETCVG  264
LjnarY(WP_010530758.1)       MYLPRICEHCLNPSCVASCPSGALYKRDEDGIVLVDQEACRGWRFCMSGCPYHKVYYNWNTHKAEKCNFCYPRTEAGLPTICSETCVG  263

HpnarY                       RIRYLGVLLYDADRIEEVASSPDERDLYHRQCEIFLDPNDPEVIAQAKRDGIQDNVIKAAQASPVYKMAIDWGLALPLHPEYRTLPMV  352
SsnarY(WP_013172229.1)       RMRYMGVMLYDADRVEEMANTENEQDLYHKQLEIFLDPNDPAVYEQAVRDGIPADVLEAAKASPIKKMITDWKVALPLHPEYRTMPMV  350
AanarY(WP_008166145.1)       RIRYLGVLLYDADQIENAASTASEQDLYEEQLGLFLDPHSPQVIAAAREQGIPESWLEAARNSPVYKMAVEWKVAFPLHPEYRTLPMV  352
EcnarY(WP_000702569.1)       RIRYLGVLLYDADRIEEAASTEREVDLYERQCEVFLDPLDPSVIEEALKQGIPQNVIEAAQRSPVYKMAMDWKLALPLHPEYRTLPMV  352
LjnarY(WP_010530758.1)       RIRYIGVVLYDADKVKEAASVEDPQALYESQLSVFQDPFDPEVIEEARKAGINDEWIESAQKSPVYKMAMKWKIALPLHPEYRTLPMV  351

HpnarY                       WYVPPLSPIQSAAEAG-HVEFD----GILPKIESLRIPVKYLANLLTAGEEEPVVLALKRLMAMRIYMRGKHVDG---APNAEVLDAV  432
SsnarY(WP_013172229.1)       WYIPPLSPIMNLMDDA---DTKSGADQLFPAIDEMRIPIEYLAGLLTAGDTSHIRHVLKKMAVMRDYMRTKQTG--RAFDG-KVTEEL  432
AanarY(WP_008166145.1)       WYIPPLSPIQSAAESGKMPQRTVGKSGVIPDVSSLRIPVRYLANLLTAGKEAPVLQALERMLAMRAYKRSETVHG---ERDTALLDQV  437
EcnarY(WP_000702569.1)       WYVPPLSPIQSYADAGGLPKSE----GVLPAIESLRIPVQYLANMLSAGDTGPVLRALKRMMAMRHYMRSQTVEG---VTDTRAIDEV  433
LjnarY(WP_010530758.1)       WYVPPLSPIMNHITNE---DDLS-TDGYIPAIDQMRIPVEYLASILAADGTKVIRKVLLKLTAMRVHMRGKTVGGIDEFRQSELLKEA  435

HpnarY                       GLSVAQVEEMYRYLAIANYEDRFVIPTSHREMATEAFPERGGCGFTFGDGCHG-ESQPNLFNGRKQTSVLVKPVEVFDPQPQLEESRH 519
SsnarY(WP_013172229.1)       GLTEQDIEDMYRLLAIAKYDDRFVIPVSEKEYADDLYQMQGECGLDFD---GGP-GSCSVLQNQRG---------------------- 494
AanarY(WP_008166145.1)       GLSEETVQDMYRIMAIANYEDRFVVPSSHKEVVEDSFNEKGSCGFTFGNGCSGGVSEGTLFGRKPQGS--EIFIEMPKSRKKAVSA-- 521
EcnarY(WP_000702569.1)       GLSVAQVEEMYRYLAIANYEDRFVIPTSHREMAGDAFAERNGCGFTFGDGCHGSDSKFNLFNSSR-----IDAINITEVRDKAEGE-- 514
LjnarY(WP_010530758.1)       NTTPEELEDMARLLGVAKYNERFVIPTGRREMDDNLYYEQGACSLEDL---APPEGMASLIGGNR---------------------- 497

HpnarY                       D 520
SsnarY(WP_013172229.1)       -   -
AanarY(WP_008166145.1)       -   -
EcnarY(WP_000702569.1)       -   -
LjnarY(WP_010530758.1)       -   -
```

nirD

```
HpnirD                      -MTATAMKKEMDMADVHNSEAFENHASENNTA--WQKVCTKDDLVAFSGIAAWLETAEGPAQVAIFYLPG-LGQGKGDELFALDHH   82
CanirD                      -MTSPALATTFSTPLSSPSSATQDTSAATQSPLRWVTLCQREDLVPGSGVAAWLEADGQARQIALFTLPATVDS-DDLTLFAIDHN   84
HvnirD(WP_125746047.1)      -MTATALK-----------------HTVSTDT---WQPLCTKADLVAFSGVAAWLNTPEGPAQVAIFYLPKQHPQGHEKELYALDHF   66
AhnirD(WP_092568117.1)      MTTATALK-----------------TETDTMT---WQTICSKADLVAFSGVAAWLDTSDGPAQVALFYLPG-----HDQELYAVDHH   62
HhnirD(WP_095602547.1)      -MTATALK-----------------HTVSTDT---WQPLCAKADLVAFSGVAAWLNTSEGPAQVAIFYLPRQRSQGQEKELYAIDHF   66

HpnirD                      DPFSNANVIARGIVGDLKGSAVVASPIYKQHFRLEDGQCLEDEDVKLRTWKVEFKGDEVWVEG-------  145
CanirD                      DPVSGANVIARGLLGDHAGEPLVISPLYKQRYRLSDGQCLDDDTLALTVWPVRLAGGEVQVQCQRTDDAC  154
HvnirD(WP_125746047.1)      DPFSNANVIARGIIGDVKGNPVVASPLYKQHFRLEDGQCLEDENIKLRTWKVAFRGEEVWVAG-------  129
AhnirD(WP_092568117.1)      DPFSRANVIARGIVGDLKGQPVVASPIYKQHFRLEDGQCLEDESVQLRTWKVSFKGDEVCIEV-------  125
HhnirD(WP_095602547.1)      DPFSNANVIARGIIGDIKGSSVVASPLYKQHFRLEDGQCLEDENIKLRTWKVAFRGEEVWVQG-------  129
```

nirS

```
HpnirS                   MKKRNRYLRPLAVAVTAASLGLGIVHAFEDDPNQDQEAERLYRGTDSPVDAASANAVRTPGAPDLTEEEFERAKEIYFQRCAGCHG   86
PbnirS(MCG8523864.1)     MRVTKLMIKPLALAVAITSLGVMNAQAAPKDPDK---STMAYEGTPSAVEAESAKIVRSPGAPDLTDAEFEKAKQIYFQRCAGCHG   83
MsnirS(GBO88462.1)       MRVSNLLFKPLALAVAVASFGVMTVQATPKDPDP---STSAYEGTASAVDPESARVVRTPGAPDLTDAEFEKAKQIYFERCAGCHG   83
MpnirS(WP_091705256.1)   MRVTKMMIKPLAMAVAFASIGVMTAQAAPKDADK---SSVAYEGTPSTVDPSSAKSVRTPGAPDLTEAEFEKAKQIYFERCAGCHG   83
MnnirS(WP_227548888.1)   ------MIKPLALAVAITSLGVMNAQAAPKDPDK---STMAYEGTPSAVDAESAKIVRSPGAPDLTDAEFEKAKQIYFQRCAGCHG   77

HpnirS                   VLRKGATGKPLTPDITQELGLDYLKVFINYGSPAGMPNWGTSGDFSEEEVELMAKYVMHEPPVPPEFGMAEMQESWEVVVPPEERP   172
PbnirS(MCG8523864.1)     VLRKGATGKPLTPDITQERGIDYLKAFISYGSPAGMPNWLTSGDFDEETVELMAKYIMHEPPMPPEFSLADMKETWEVIVPPEDRP   169
MsnirS(GBO88462.1)       VLRKGATGKPLTTDITQERGIDYLKAFISYGSPAGMPNWLTSGDLTEEEVELMAKYVMHEPPQPPEFSLEDMKATWEVVVPPEERP   169
MpnirS(WP_091705256.1)   VLRKGATGKPLTPDITQERGIDYLKAFISYGSPAGMPNWLTSGDFDEETVELMAKYIMHEPPQPPEFSLADMKETWEVIVPPEDRP   169
MnnirS(WP_227548888.1)   VLRKGATGKPLTPDITQERGIDYLKAFISYGSPAGMPNWLTSGDFDEETVELMAKYIMHEPPQPPEFSLADMKETWEVIVPPEDRP   163

HpnirS                   TEQMNDLDLENMFSVTLRDAGQIALIDGGSKEIVDILDTGYAVHISRISASGRYLFVIGRDAKVDMIDMWMEEPGIVATIKVGMEA   258
PbnirS(MCG8523864.1)     TKQMNDLDLENLFSVTLRDAGQIALIDGDSKEVVKIIDTGYAVHISRMSASGRYVMVIGRDALINMIDLWMEEPKTVAKIKVGMEA   255
MsnirS(GBO88462.1)       TKQMNDLNLKNLFSVTLRDAGQVALIDGDTHEIVHIIDTGYAVHISRMSASGRYVFVIGRDALVNMIDLWMEEPNTVAKIKVGMEA   255
MpnirS(WP_091705256.1)   TKQMNDLNLENIFSVTLRDAGQIALIDGDSKEVVKIIDTGYAVHISRMSASGRYVMVIGRDALINMIDLWPEEPQTVAKIKVGMEA   255
MnnirS(WP_227548888.1)   KKQMNDLDLENLFSVTLRDAGQIALIDGDSKEVVKIIDTGYAVHISRMSASGRYVLVIGRDALINMIDLWMEEPQTVAKIKVGMEA   249

HpnirS                   RSVETSKFEGYEDRFAIAGAYWPPQYVIMDGETLEPLRIVSTRGMTVGTQEYHPEPRVAAIVASHQHPEFIVNVKETGKVQLVNYE   344
PbnirS(MCG8523864.1)     RSVETSKYKGWEDKLAIAGTYWPPQFVIMDGDTLEPKKIVSTRGMTVGDQEYHPEPRVAAIVASHAHPEFIVNVKETGKIMLVNYE   341
MsnirS(GBO88462.1)       RSVETSKYKGWEDKLAIAGTYWPPQFVIMDGKTLEPKKIVGTRGMTVDTQEYHPEPRVAAIVASHKHPEFIVNVKETGKIMLVNYE   341
MpnirS(WP_091705256.1)   RSVETSKYKGWEDKLAIAGTYWPPQFVIMDGQTLEPKKVVGTRGMTVDPQEYHPEPRVAAIVASHAHPEFIVNVKETGKIKLVNYE   341
MnnirS(WP_227548888.1)   RSVETSKYKGWEDKLAIAGTYWPPQFVIMDGDTLEPKKIVSTRGMTVSTQEYHPEPRVAAIVASHAHPEFIVNVKETGKIMLVNYE   335

HpnirS                   NLDALSMVEIDTSRFLHDGGWESSGRYFMTAANESNQIVVIDAQDRELEAIVDVGQTPHPGRGANFVDPEFGPVWATSHLGDNTIQ   430
PbnirS(MCG8523864.1)     DMENMNITSIDAAKFLHDGGWDASMRYFLTAANNSNKIAVVDAQDRNLEAIVDVGKIPHPGRGANFVDPEHGPVWATSHLGDQTIQ   427
MsnirS(GBO88462.1)       DLDNLSVTNIDAARFLHDGGWDSSMRYFLTAANQSDKIAVVDSQDRNLEAMVDVGKIPHPGRGANFVDPEHGPVWATSHLGDPTIQ   427
MpnirS(WP_091705256.1)   DLDNMSITTIDAARYLHDGGWDASMRYFLTAANNSNKIAVVDAQDRNLEAMVDVGKIPHPGRGANFIDPEHGPVWATSHLGDNTIQ   427
MnnirS(WP_227548888.1)   DMENMNITSIDAAEYLHDGGWDASMRYFLTAANNSNKIAVVDAQDRNLEAIVDVGKIPHPGRGANFVDPEHGPVWATSHLGDQTIQ   421

HpnirS                   LIGTDPEEYPENAWRVVRTLEGQGGGSLFIKTHPESSNLYVDTPLNPGEEISQSVAVYDLNDLEAGYEVLPIAEWADVGEGPKRVV   516
PbnirS(MCG8523864.1)     MIGTDPKGHPDKAWKVVRTVDGQGGGSLFVKTHPESTNLYVDTALNPAEAVSQSVAVFDINNFDAGYEVLPIADWADLGEGPKRVV   513
MsnirS(GBO88462.1)       LIGTDPEGHPDKAWKVVRTLNGQGGGSLFIKTHPKSTNLWVDTALNPAEAVSQSIAVFDINNLDAGYDVLPIAEWANLGEGPKRVV   513
MpnirS(WP_091705256.1)   MIGTDPEGHPDKAWKVVRTVEGQGGGSLFVKTHPESDNLWVDTALNPNEAISQSIAVFDINDFDAGYEVLPIAEWADLGEGPKRVV   513
MnnirS(WP_227548888.1)   MIGTDPEGHPDKAWKVVRTVDGQGGGSLFVKTHPKSKNLWVDTALNPAEAVSQSVAVFDINNFDAGYEVLPIAEWAELGEGPKRVV   507

HpnirS                   QPEFNNAGDEVWFSVWNAMDQESALVIVDDTTRELKAVIKDERLVTPTGKFNVLNTQNDIY   577
PbnirS(MCG8523864.1)     QPEYNKAGDEVWFSVWNAQDKKSAIVVVDDKTRKLKKVIKGDYLVTPTGKFNTYNTQHDVY   574
MsnirS(GBO88462.1)       QPEYNVEGDEVWFSVWNTQDKKSALVVVDDKTRKPKKVITDERLVTPTGKFNVYNTQHDVY   574
MpnirS(WP_091705256.1)   QPEYNKAGDEVWFSVWNTQDKNSAIVVVDDETRELKKVLKGDYMVTPTGKFNTYNTQHDVY   574
MnnirS(WP_227548888.1)   QPEYNKAGDEVWFSVWNTQDKKSAIVVVDDKTRKLKKVIKGEYMVTPTGKFNTYNTQHDVY   568
```

norB

```
HpnorB                MKYETQKVALPFFAVAMALFTLQIVFGLLAATVYAWPNFMAEVMPFHIMRVSHTNLLIVWLLIGFMGCTYYLMPEEAEQEIHSPNLAY  88
AenorB(WP_011629854.1) MKYETQRVAYPFFLVAMALFALQITFGILAGTVYVLPNFLAEAMPFHIIRTSHTNLLIVWLLIGFFGCTYYLIPEETEHELYSPKLAY  88
PmnorB(WP_206559974.1) MKYESQRVALPFFAVAMALFVLQVVFGILAATVYVLPEFLAEAMPFHIMRMSHTNLLIVWLLIGFMGSTYYLLPEEAEQDIHSPLLAY  88
HbnorB(MCB1729806.1)   MKYETQRVAYPFFVVAMALFTLQVVFGIMAATVYIMPEFLSEAMPFHIMRMSHTNLLIVWLLMGFMGSTYYLMPEEAETEIHSPMIAY  88
TtnorB(WP_018233944.1) MKYETQKVALPFFMVAMALFALQLIFGLLGAIAYVAPNFLAEAMPFNIMRTSHTNLLLVWLLIGFMGCTYYLMPEEAEHEIYSPKIAY  88

HpnorB                IQLAIFAFAGAAALVGYQFGIHEGREFLEQPFWVKILITISFLMFLFNTSMTLLKGRKTAINLVLMLGLWLAAVFWLFAFYNPTNLAV 176
AenorB(WP_011629854.1) IQLGIFAFAGAAALVGYQFGIHEGRHYLEQPFWVKSLLTLSFLMFLFNTTMTLIKGRRTAVSLVLMLGLWLAAIFWMFAFYNPSNLAL 176
PmnorB(WP_206559974.1) LGLAIFAVAGAGALLSYQFGIHEGREFLEQPLWCKVLITVSFLMFLYNTTRTLLAGRKTAINLVLMLGLWLAAVFWLFAFYNPSNLSL 176
HbnorB(MCB1729806.1)   VQLAIFAVAGAGALLSYQLGIHEGREFLEQPLWSKILITVSFLMFLYNTSMTLIKGRKTAINLVLMLGLWLAAVFWLFAFFNPGNLAV 176
TtnorB(WP_018233944.1) IQLAIFAFAGAAALIGYQFGIHEGRHYLEQPLWVKVLITISFLMFLFNTSMTMINGRRTAINMVLMLGLWLAAIFWLFAFFNPANLAL 176

HpnorB                DKLYWWWVVHLWVEGVWELIMASLLGYLLIKMTGVDREVIEKWLYIIVGLALFSGLLGTGHHYYWIGAPGYWQPIGSIFSTLEVAPFF 264
AenorB(WP_011629854.1) DKLYWWWIVHIWVEGVWELIMAALLGYLLIKLTGVDREIIEKWLYVIVGLSLFSGLLGTGHHYYWIGTPGYWQPIGSIFSTLEVIPFF 264
PmnorB(WP_206559974.1) DKLYWWWIVHVWVEGVWELIMASLLAYLLIKMTGVDREVIEKWLYVIIGLALFSGLLGTGHHYYWIGTPGYWQPLGSIFSTLEVAPFF 264
HbnorB(MCB1729806.1)   DKLYWWWVVHLWVEGVWELIMASLLAYLLIKMTGVDREIIEKWLYVIIGLALFSGLLGTGHHYYWIGAPEYWQPLGSIFSTLEVLPFF 264
TtnorB(WP_018233944.1) DKLYWWWIVHIWVEGVWELIMASLLGYLLIKMTGVDREIIEKWLYVIVGLSLFSGLLGTGHHYYWIGTPGYWQPIGSIFSTLEVIPFF 264

HpnorB                AMVVFAFTMFWKGSRNHPNKAAMLWALGCPTIAFFGAGVWGFMHTLSFVNYYSHGTQVTAAHGHLAFYGAYVMLIILGVITYAMPQIRR 352
AenorB(WP_011629854.1) AMVVFAFYMFWKGSRNHPNKAAMLWTLGCAVIAFFGAGVWGFMHTLSFINYYTHGTQITAAHGHLAFFGAYVMLVLALISYAMPQMRR 352
PmnorB(WP_206559974.1) AMVMFAFYMFWKGRRNHPNKSAMLWTLGSATVAFFGAGVWGFMHTLSFVNYYSHGTQVTAAHGHLAFYGAYVMMVLAIITYAMPQLRR 352
HbnorB(MCB1729806.1)   AMVMFAFFMFWKGRRNHPNKAAMLWTLGSATLAFFGAGVWGFMHTLSFVNYYSHGTQVTAAHGHLAFFGAYVMTVLAIITYAMPQLRR 352
TtnorB(WP_018233944.1) AMVVFAFYMFWKGSRNHPNQAAMLWTLGCATIAFFGAGVWGFMHTLSFINYYTHGTQITAAHGHLAFYGAYVMLVLAIITYAMPQLRR 352

HpnorB                VQPYNQVLNMWGFWIVTSAMCFMTFTLTFAGVVQTHLQRVLGMNYMEVQDQLGLFYIMRLGAGVAVAVGAIMLIYSFFGPAREQVPAG 440
AenorB(WP_011629854.1) VQPYNQVMNMWSFWIMTSAMCFMTFTLTFAGVVQTHLQRVLGMNFMEVQGQLDMFYVMRLGAGVAVAVGLVLFLWSIYGRTREQVPQT 440
PmnorB(WP_206559974.1) MQPYNQILNMWSFWMMTSAMCFMTFTLTFAGVVQTHMQRVMGINYMEVQAQLGLFYAMRLGAGAVVAVAALLYLYAVFGPAREQLPAT 440
HbnorB(MCB1729806.1)   VQPYSQILNMWSFWLMTSAMCFMTFTLTFAGVVQTHLQRVMGMNYMEVQDQLQLFYYMRLGAGVVVAIAAVLYLIAIFGPTKSQVSAT 440
TtnorB(WP_018233944.1) VQPYNQVLNMWAFWIMTSAMAFMTFTLTFAGVVQTHLQRVMGMNFMEVQSQIEMFYVMRLGAGVFVIIAALMFFYAVFGPAREQVPAY 440

HpnorB                GTQITAGAGSV 451
AenorB(WP_011629854.1) -ANVPGTA--- 447
PmnorB(WP_206559974.1) SQTALGTT--- 448
HbnorB(MCB1729806.1)   TESQLGTATG- 450
TtnorB(WP_018233944.1) -AKASATAE-- 448
```

norC

```
HpnorC                   MADGLTKSAARNIFYGGSLFFFLLFAALTAHSHWYMVNVSTDSEGLTDSVKHGKEVWEKNMCINCHTIMGEGAYFAPELSNVWERYGG   88
HsnorC(WP_027950149.1)   MMESLSRSAARNIFFGGSLFFFLAFAGLTAHSHWYMVNVSTDKEGLTESVAAGKHVWEKHSCINCHSLIGEGAYFAPELGNVWTRYGG   88
CcnorC(WP_183317601.1)   MADSLTKSAARNVFYGGSLFFFVIFVGLTAHSHYFMVNVSTDKAGLTDAVARGKHVWEKHSCINCHTLLGEGAYFAPELGNVWDRWGG   88
TnnorC(WP_043740377.1)   MMEGLSRAAARNIFLGGSLFFFVVFVALTAHSHWYIVTQSTDHAGLTESVKHGKEVWEEHSCINCHTLLGEGAYFGPELGNVWVRYGG   88
AanorC(WP_128291034.1)   MSERFTKSAARNIFYGGSIFFFVAFVGLTAESHWHIVSQSTDTATLTPSVAHGKRVWEKNACIDCHTLLGEGAYFAPELGNVWVRYGG   88

HpnorC                   LENPEAARAGINAWIRAQPLGIEGRRQMPAFDFSEKDMNSLIDFLEWINSINDQDWPPHPAG 150
HsnorC(WP_027950149.1)   RENPDAARAGIKGWIKAQPLNVPGRRQMPKFDLSEEELDNLVDFFRWADSINTQGWPPHPVG 150
CcnorC(WP_183317601.1)   TEDPDTAREMLKAWMQSQPTGIEGRRQMPQFNLSDQELNDLADFLEWTGRIKTQNWPPNKAG 150
TnnorC(WP_043740377.1)   PDSPEGARAGIKAWMQIQPTGIPGRRQMPNFDLTDAELDALVDFFEWMSRINTQGWPPHVAG 150
AanorC(WP_128291034.1)   RDDPEIAREGLKAWIRSMPTGIEGRRQMPHFDLSEKELNDLIDFLEWTSRIDTQDWPPNDAG 150
```

nosZ

```
HpnosZ                  MSDKKDHIQD--------IGRRHFLRNTAVTGVAGAG---LASGFGAGALLNSRQVQAAGANG--IDVAPGELDDYYGFWSGGHSGEV  75
MnnosZ(WP_222448800.1)  MKKRDDLTKDTPEVSEGGLSRRRFMGAAALAGVAGAT------GLGTSVMSRETWAAAAEEARNKAHVAPGELDEYYGFWSGGHQGEV  82
ManosZ(WP_230707954.1)  MKKRDDLTKDLQELPESGQSRRRFMGAAALAGVAGAT------GLGTAVMSREAFAAAAEEARNKYVIHPGELDEYYGFWSGGHSGEV  82
MgnosZ(WP_091987576.1)  MRKKDDLIKGSPALPESGQSRRRFMGAAALAGVAGAT------GLGSAVMSREAFAAAAEEARNNWLIHPGELDEYYGFWSGGHSGEV  82
HcnosZ(WP_011397207.1)  MKDADKSSHTTPDARDSGISRRGFLGGAAVTGVSAVTGMAAMTGFGSSIMSPESWAAAAKTAHQKASVEPGELDEYYGFWSGGHSGEV  88

HpnosZ                  RVLGVPSMRELMRIPVFNVDSASGWGISNESKRILGESSRFLNGDCHHPHISMTDGSYDGKYLFINDKANTRVARIRLDVMKTDKITT  163
MnnosZ(WP_222448800.1)  RVLGVPSMRELMRIPVFNVDSATGWGITNESKEILGGDQQYLNGDCHHPHISMTDGRYDGKYLFINDKANTRVARIRLDIMKTDKITH  170
ManosZ(WP_230707954.1)  RVLGVPSMRELMRIPVFNVDSATGWGISNESKDVLGHDNTHLNGDSHHPHISMTDGRYDGKYLFINDKANTRVARIRLDIMKCDKITT  170
MgnosZ(WP_091987576.1)  RVLGVPSMRELMRIPVFNVDSATGWGLTNESKEVLGDSSRFSNGDSHHPHISMTDGRYDGKYLFINDKANTRVARIRLDIMKCDKITT  170
HcnosZ(WP_011397207.1)  RVLGVPSMRELMRIPVFNVDSATGWGLTNESKQVLGESARFSNGDAHHPHVSMTDGRYDGKYLFINDKANTRVARIRLDIMKCDKITT  176

HpnosZ                  IPNVQAIHGLRLQKVPYTKYVFANAELPIPQINDGRDLENPDNYFTMFNAIDAESMDVAWQVIVDGNLDNTDADYTGRFVASTCYNSE  251
MnnosZ(WP_222448800.1)  IPNVQAIHGLRLQKVPKTNYVFCNAEFVIPQPNDGTDFS-LDNSYTMFTAIDAETMDVAWQVIVDGNLDNTDADYTGKYAASTCYNSE  257
ManosZ(WP_230707954.1)  IPNVQAIHGLRLQKVPKTKYVFCNAEYVIPHPNDGSDTS-LESSFTMFNAVDAETMEVAFQVIVDGNLDNTDADYTGKYACSTCYNSE  257
MgnosZ(WP_091987576.1)  IPNVQAIHGLRLQKVPKTKYVFCNAEYVIPHPNDGSDTS-LENSFTMFNAVDAETMEVAFQVIVDGNLDNTDADYTGKYACATCYNSE  257
HcnosZ(WP_011397207.1)  IPNVQAIHGLRLQKVPRTKYVFCNAEFVIPQPNDGTDMENLENHYTMFNAVDADTMEVAWQVIVDGNLDNTDADYTGKYVASTCYNSE  264

HpnosZ                  KGMTLTETMRNERDWVVVFDVEAIEAAVAAGNYKTLGESSVPVLDGRKGSQLTRYIPIPKNPHGVNTSPDGKYFIANGKLSPTCSIIA  339
MnnosZ(WP_222448800.1)  RAVDLAGTMRNDRDWVVVFNVERIAAAVKAGNFKTIGDSKVPVVDGRGKSEFTRYIPVPKNPHGLNTSPDGKYFIANGKLSPTVSVIA  345
ManosZ(WP_230707954.1)  KALDLAGTMRNDRDWAVVFNIERIEQAVKNGNYKTLGDSKVPVVDGRAGSELTRYIPVPKNPHGLNTSPDGKYFIANGKLSPTATIIA  345
MgnosZ(WP_091987576.1)  KALDLAGTMRNDRDWAVVFNIARIEQAVKNGNYKTLGDSKVPVLDGREGSEFTRYIPVPKNPHGLNTSPDGKYFIANGKLSPTVSIIA  345
HcnosZ(WP_011397207.1)  KGLLLTDTMRNERDWVVVFNVPRIEAAIQAGNFKTIGDAKTPVVDGRHGSELTRYIPVPKNPHGLNTSPDGKYFMANGKLSPTVSIIA  352

HpnosZ                  IDKLPELFDDSIE-PRDTIVGEPELGLGPLHTTYDGRGNAYTTLFIDSQVAKWNIEDAIRHYNGEDVNYLRQKLDVHYQPGHNHASLT  426
MnnosZ(WP_222448800.1)  IDKLDDLFEDKIE-LRDTIVAEPELGLGPLHTTFDGRGNAYTTLFIDSQVCKWNIADAIKHYNGDKVNYIRQKLDVQYQPGHNHASLT  432
ManosZ(WP_230707954.1)  IDKLDDLFDGKIE-PRDTVVGEPELGLGPLHTTFDGRGNAYTTLFIDSQVAKWNIADAIRHYNGEDVNYIRQKLDVHYQPGHNHASLT  432
MgnosZ(WP_091987576.1)  IDKLDDVFDNKIE-PRDAVVAEPELGLGPLHTTFDGRGNAYTTLFIDSQVAKWNIADAIRNYNGEKVDYIRQKLDVHYQPGHNHASLT  432
HcnosZ(WP_011397207.1)  IDKLDDLFDGKFKDERDVIVGEPELGLGPLHTTFDGRGFAYTTLFIDSQVAKWNIAEAIRAYNGEKVNYIKQKLDVQYQPGHNHASLT  440

HpnosZ                  ESRDADGKWLVVLSKFSKDRFLPVGPLHPENDQLIDISGEEMKLVHDGPTYAEPHDCILVRADQINPSRVWTRDDPFFAETVAMAEAD  514
MnnosZ(WP_222448800.1)  ESRDADGKWLVVLSKFSKDRFLPVGPLHPENDQLIDISGEEMKLVHDGPTYAEPHDCILVRRDQIKTKKIYERNDPYFASCRAQAEKD  520
ManosZ(WP_230707954.1)  ESRDADGKWLVVLSKFSKDRFLPVGPLHAENDQLIDISGEEMKLVHDGPTFAEPHDCILVRRDQIKTKKIYDRDDPFFASAVEQARKD  520
MgnosZ(WP_091987576.1)  ESRDADGKWLVVLSKFSKDRFLPVGPLHAENDQLIDISGEEMKLVHDGPTFAEPHDCILVRRDQIKTKKIYTRDDPFFATARAQAEKD  520
HcnosZ(WP_011397207.1)  ESRDADGKWLVVLCKFSKDRFLPAGPLHPENDQLIDISGDEMALVHDGPTYAEPHDCILVRRDQIRPLKIWSRDDPFFASAVAQAKKD  528

HpnosZ                  GVNLEGESKVIREGNKVRVYMTSIAPQFGLTEFKVKQGDEVTVVITNLDQVEDLTHGFCMVNHGVQMEISPQQTASVTFTADKAGVHW  602
MnnosZ(WP_222448800.1)  GVTLESDNKVIRDGNKVRVYMTSVAPQYGMTEFKVKEGDEVTVYITNLDMVEDVTHGFCMVNHGVSMEISPQQTASVTFTAGKPGVYW  608
ManosZ(WP_230707954.1)  GVTLEADNKVIRDGNKVRVYMTSIAPQYGMTEFKVKQGDEVTVYVTNLDTIEDVTHGFCMVNHGVSMEISPQQTSSVTFKADRPGVHW  608
MgnosZ(WP_091987576.1)  GVTLEADNKVIRDGNKVRVYMTSIAPQYGMTEFKVKQGDEVTVYVTNLDTIEDVTHGFCMVNHGVSMEISPQQTASVTFKADRPGVHW  608
HcnosZ(WP_011397207.1)  GITLETDNKVIRDGNKVRVYMTSVAPIYGLTEFRVKQGDEVTVYITNMDMVEDVSHGFCMVNHGVSMEISPQQTSSVTFVASEPGVHW  616

HpnosZ                  YYCNWFCHAMHMEMTGRMLVEPA  625
MnnosZ(WP_222448800.1)  YYCNWFCHALHMEMGGRMLVEKA  631
ManosZ(WP_230707954.1)  YYCNWFCHALHMEMRGRMIVEKA  631
MgnosZ(WP_091987576.1)  YYCNWFCHALHMEMRGRMIVEKA  631
HcnosZ(WP_011397207.1)  YYCNWFCHALHMEMRGRMIVEKA  639
```

ppc

```
Hpppc                     MSHDLHESLRDNVRILGDSLGRTIADDLGQAFVDKIETIRAHAKRGRQG---DSLQQRELIEYLRELPEQDLLPVTRAFNQFLNLANIA  86
Cappc                     MSHDLHESLRDNVRILGDNLGQTIAADLGDDFVARIERIRALAKQGQQG---DQAARHELIDYLRDIPDGDLLPVTRAFNQFLNLSNIA  86
Osppc(WP_019935846.1)     --MDNHAALRANVGLLGQLLGDAIRDHHGQAFLDKIETIRQLAKSARQG---NDNDQQRLLTTLKQLSDDELLPVARAFSQFLNLANVA  84
Mdppc(WP_017223576.1)     -MSDKYAALRSNVGLLGQVLGNTIKDHLGEEFLDKIETIRQLAKSSRAG---NDKDRAQLLTVLRGLSDDELLPVARAFSQFLNLANIA  85
Plppc(WP_003189102.1)     MS-DIDARLREDVHLLGELLGNTIREQYGEAFLEKIEQIRKGAKADRRGAVSEKTAGDELSASLNQLQEQELLPVARAFNQFLNLANIA  88

Hpppc                     EQHYRARFRRVEDYKPGSQPVLGELLARAEQAGNSPRKLVETLANMRVELVLTAHPTEVIRRTLIQKYDAIDECLTAIESSEDYPERGT  175
Cappc                     EQHYRGRFKRVEDYRPGSQPDLGELITRIRERGFSAQKIVEELSAMRVELVLTAHPTEVIRRTLIQKYDAIEGCLTQMEGACDRPEQAT  175
Osppc(WP_019935846.1)     EQFHTISRGYRKDAG---PNPLDEVFARLKGANVSQADIRGAVTNLDIDLVLTAHPTEVTRRTFIHKHVQLNDCLAAMEL-DLPEDEKT  169
Mdppc(WP_017223576.1)     EQFHTTSRNCADNVCV--PDSIDELFAKLNNSEISEEDVLSAVKNLNIDLVLTAHPTEITRRTLIHKHGAINKCLSQLELTELSDQERN  172
Plppc(WP_003189102.1)     EQYQLIHRRDESQPAPFESRVLPELLARLQSEGHSNESLARQLARLDIELVLTAHPTEVARRTLIQKYDAIAAQLALQDHRDLTTAERE  177

Hpppc                     RAQGRLEELISQAWHTDEIRHERPTPVDEAKWGFAVIENSLWQAVPDFHRDLDNLLLDTAGERLPLDAAPIRYASWMGGDRDGNPNVTA  264
Cappc                     LARARLTELISQAWHTDEIRHERPTPVDEAKWGFATIENSLWQAVPDFHRQLDEMTLDAAGERLPLDAAPICFASWMGGDRDGNPNVTA  264
Osppc(WP_019935846.1)     ELLARIDQLITQAWHSNEIRSQRPTPVDEAKWGFAVIENSLWPALPRFLRQLDQRMERELGERLPLNAAPVRFTSWMGGDRDGNPFVTA  258
Mdppc(WP_017223576.1)     VFMTRIEQLVSQAWHTNEIRTVRPTPVDEAKWGFAVVENSLWDAVPNFLRRLDTRLQDRLDYQLPIDASPVKFTSWMGGDRDGNPFVTS  261
Plppc(WP_003189102.1)     QIRERLQRLIAEAWHTEEIRRVRPTPVDEAKWGFAVIEHSLWHAIPNYLRRADQALHAATGLRLPLEAAPIRFASWMGGDRDGNPNVTA  266

Hpppc                     RVTREVLLLGRWMAADLYLRDLEQLKTELSMWKANSALKAEAGDVAEPYREVLKRLLAKMESTRDWAKAELEGRNYDGGPIIETRDQLY  353
Cappc                     SVTRTVLLLGRWMAADLYLRDLEQLKSELSMWKCTAALRAEAGDVAEPYREILRRLILRMESTRDWAKAGLDGKRHEGVDIIETREQLY  353
Osppc(WP_019935846.1)     RVTEEVLLLGRWMASSLFLNDIQELVSELSMSDASTELRAASGDSDEPYRAVLRQLREDLRETLLHLNARVQGQKTDARDLITQTEQLR  347
Mdppc(WP_017223576.1)     KVTEEVLLTSRWMAIHLFLKDVTHLTSELSMNDCDDTLRALVGESHEPYRVLLKKLRSELTETLASLTAQLKGESTESRDIITKTAQLR  350
Plppc(WP_003189102.1)     PVTREVLLLARWMAADLYLRDIDQLASELSMQQASPALHAKVGDSVEPYRALLKQLRERLRATRQWAHTSLTSTTPAPADVLQNNRDLL  355

Hpppc                     SPLLACYRSLCDVGLDTIANGALLDTLRRVAVFGVTLTKLDLRQEASRHAQVIEELTSALGLGHYQEWDEAKRQEFLLEELASRRPLIP  442
Cappc                     TPLLACYRSLCDVGLDTIANGALLDTLRRVACFGVTLTKLDIRQESTRHAQVFDELSQTLGLGSYFEWDEQQRCDFLTKELAGRRPLIP  442
Osppc(WP_019935846.1)     QPLELCYHSLQQCGLGKIADGLLLDVIRKVACFGIHLLKLDIRQDGERHAQAIAELTRYLGLGDYGEWSEADKQAFLLNELNSRRPLLP  436
Mdppc(WP_017223576.1)     QPLLACYNSLNACGMAVIAKGAILDTIRRVECFGVSLVKLDIRQDSDRHTDVIAEVTRYLGLGDYAQWSEQDKQAFLLQELNNKRPLLP  439
Plppc(WP_003189102.1)     APLQLCYQSLHECGMGVIADGPLLDCLRRAVTFGLFLVRLDVRQDSSRHSAAMTEITDYLGLGRYEDWDEEARISFLTQELTNRRPLLP  444

Hpppc                     RRWECSAESREVLDTFKVIAQEHSEALGTYIISMAAEPSDVLTVALLMKEVGGQVTLPIAPLFETLNDLDHAGDVVDQLLAIPGYRSLI  531
Cappc                     RHWECSTDTREVLDTFKVIAREHREALGTYIISMAGQASDVLAVALLMKEIGGDVRLPIAPLFETLDDLDRSGPVIDTLLGMPIYREQA  531
Osppc(WP_019935846.1)     RQWQPSADTQETLDTCRVISRHPQDAFGIYIISMAGEPSDVLAVQLLLKECGVDFAMPIAPLFETLDDLNQGAAVIERLYQQPWYRGYM  525
Mdppc(WP_017223576.1)     ICWQPSADVKEVLDTCRTVAKQDPESLGIYIISMARKASDVLAVQLLLKETGCPFRLPVAPLFETLDDLNNAGTVTASLLAIDWYKNYI  528
Plppc(WP_003189102.1)     GYFKPSADTAEVLNTCKEIAAAPAASLGSYVISMAGAASDVLAVQLLLKESGVQRPMRVVPLFETLADLDNAGPVMERLLQLPGYRARL  533

Hpppc                     DDRQEVMIGYSDSAKDAGQLAAAWAQYRAQESLVNVCNRHGVDLTLFHGRGGTVGRGGGPAHAAILSQPPGSVNGSLRVTEQGEMIRFK  620
Cappc                     GDRQEVMIGYSDSAKDAGQLAAAWAQYRAQETLVEVTKRHGVELTLFHGRGGTVGRGGGPAHAAILSQPPGSVTGSLRVTEQGEMIRFK  620
Osppc(WP_019935846.1)     QGRQYVMIGYSDSAKDAGMLAAGWAQYSAMEKLVAISEREKVTLTLFHGRGGSLGRGGGPARQAILAQPPGSTIGGLRVTEQGEMIRFK  614
Mdppc(WP_017223576.1)     NGHQHIMIGYSDSAKDAGVIAASWAQYRSQEALVAICEQEDINLTLFHGRGGSIGRGGAPAHAALLSQPPGSLKGGLRVTEQGEMIRFK  617
Plppc(WP_003189102.1)     QGPQEVMIGYSDSAKDAGTTAAAWAQYRAQERLVDICREQQVELLLFHGRGGTVGRGGGPAHAAILSQPPGSVAGRFRTTEQGEMIRFK  622

Hpppc                     FGQPDIALRSMEIYACAVLEATLLPPRAPEPHWREEMDQLAKVAHGAYVGVVREDPDFVPYFRAVTPEGALGRLPLGSRPTKRRQDGGV  709
Cappc                     FGLPDIASRSMEIYVCAVLEASMLPPPAPKQQWRDEMDRLAGIAHKAYVGVVREDPNFVPYFRSVTPETALARLPLGSRPAKRRQDGGV  709
Osppc(WP_019935846.1)     FGLPQVAIDSLTLYASAVLEANLLPPPEPKPEWRKLMNTMAEVSCAHYRSLIRGEPDFVPYFRAATPELELGKLPLGSRPAKRKPNGGV  703
Mdppc(WP_017223576.1)     FGLADVAEQSLNLYTSAILEANLLPPPAPEQAWRDAMDQMSADSCEEYRSYIQGHEKFVPYFRSATPELELGKLPLGSRPAKRKPNGGV  706
Plppc(WP_003189102.1)     FGLPDIAEQNLNLYLAAVLEATLLPPPPPEPAWRHLMDELAADGVRAYRAVVRENPQFVEYFRQSTPEQELGRLPLGSRPAKRR-AGGI  710

Hpppc                     ETLRAIPWIFAWTQIRLMLPAWLGSGEAFSRRLEQPGGRDVLQEMRNEWPFFGTYLDMLEMLLAKADVAIAAYYEHRLVDEPSLKALGK  798
Cappc                     ETLRAIPWIFAWTQIRLMLPAWLGSGEAFATRMQEEGGLEVLREMMNDWPFFSTYVDMLEMLLSKADLSTAAYYEQRLVDEPALKALGQ  798
Osppc(WP_019935846.1)     ESLRAIPWIFAWTQNRLMLPAWLGAHKGLEKVMA-DGHEATLHAMNQGWPFFRARLDMLEMVFLKADLGIAAYYDSVLVP-DELKALGK  790
Mdppc(WP_017223576.1)     ESLRAIPWIFAWTQNRLMLPAWLGCTAAFKNMLD-SGQRETLQEMQQQWPFFNTRLEMFEMVFLKADAWLAEYYDQCLVT-EDLWHLGK  793
Plppc(WP_003189102.1)     ESLRAIPWIFGWTQTRLMLPAWLGWEAALSKALERGEG-QLLGQMREQWPFFRTRIDMLEMVLAKADADIARLYDERLVQ-PDLLALGA  797

Hpppc                     QLRQRFASLEEAVLDILQQEKLLENTPLIRQAIDVRNPYIDPLHGLQAELLQRNRDADGAISADLSRALMVTMAGIAAGLRNTG  882
Cappc                     SLRDRLERLEKVVLEIRDQQALLEHMPLIRQAIDVRNPYIDPLHGLQAELLQRNRDADGAISAELSRALMVTMAGISAGLRNTG  882
Osppc(WP_019935846.1)     TLRDELRASTELVLKLKEKSELLASEPWIQESIKLRNPYTDPLNVLQAELLKRSRE-QGDIHPVLDQALMVTIAGIAAGMRNTG  873
Mdppc(WP_017223576.1)     ELRASLALATELIIGLTPEANLLDGQPWSKTSIELRNPYTDPLNILQAELLSRARS-NEDVNPDIEQALMVSIAGVAAGMRNTG  876
Plppc(WP_003189102.1)     HLRDLLSQACDVVLGLTGQSQLLAHSPDTLEFIRLRNTYLDPLHLLQAELLARSRQQEAAQDSPLEQALLVSVAGIAAGLRNTG  881
```
